# Zinc: Multidimensional Effects on Living Organisms

**DOI:** 10.3390/biomedicines9020208

**Published:** 2021-02-22

**Authors:** Math P. Cuajungco, Maria Soledad Ramirez, Marcelo E. Tolmasky

**Affiliations:** Center for Applied Biotechnology Studies, Department of Biological Science, California State University Fullerton, Fullerton, CA 92831, USA; mcuajungco@fullerton.edu (M.P.C.); msramirez@fullerton.edu (M.S.R.)

**Keywords:** metalloproteins, zinc transporters, metal chelators, antibiotic resistance, antimicrobials

## Abstract

Zinc is a redox-inert trace element that is second only to iron in abundance in biological systems. In cells, zinc is typically buffered and bound to metalloproteins, but it may also exist in a labile or chelatable (free ion) form. Zinc plays a critical role in prokaryotes and eukaryotes, ranging from structural to catalytic to replication to demise. This review discusses the influential properties of zinc on various mechanisms of bacterial proliferation and synergistic action as an antimicrobial element. We also touch upon the significance of zinc among eukaryotic cells and how it may modulate their survival and death through its inhibitory or modulatory effect on certain receptors, enzymes, and signaling proteins. A brief discussion on zinc chelators is also presented, and chelating agents may be used with or against zinc to affect therapeutics against human diseases. Overall, the multidimensional effects of zinc in cells attest to the growing number of scientific research that reveal the consequential prominence of this remarkable transition metal in human health and disease.

## 1. Introduction

Zinc, an essential component of life in the three domains, follows iron as the second most abundant transition metal ion in living organisms [1,2]. About 5–6% and 9–10% of proteins from prokaryotes and eukaryotes, respectively, depend on this metal to fulfill their biological functions [1]. A bioinformatics study found that over 50% of zinc-bound proteins are enzymes, and in the vast majority of them, the metal plays a catalytic role [3]. About 20% of them use zinc as a structural component, and in a small percentage, it is a regulator or substrate of enzymatic activity [3,4,5]. The requirement of zinc in such a high number of proteins illustrates its fundamental role in numerous biological processes [6,7,8,9,10,11]. The essential nature of zinc for cellular viability, together with the toxic nature of the element in higher concentrations, led prokaryotes [12] and eukaryotes [13] to evolve export and import systems to keep ionic homeostasis.

This review summarizes representative examples of zinc’s action on bacterial virulence and antibiotic resistance gene dissemination, different bacterial mechanisms to maintain zinc homeostasis, and possible uses of the metal ion in the development of novel therapeutics. It also discusses multiple roles that zinc ions play in mammalian cells, including its modulatory effect on cell signaling and cell death, in which a wide variety of therapeutic applications is continuously being researched and developed to produce an immense volume of information. Therefore, this review offers the reader a representative rather than an exhaustive description of mechanisms of action, uptake, and export as well as pharmacological uses of zinc and its chelators.

## 2. Prokaryotes

### 2.1. Zinc as Antimicrobial

Zinc is a member of the group of metals that participate in the nonspecific mechanisms of defense against infection [14,15]. The host defenses reduce trace elements’ availability to starve the infecting bacterial cells in a response known as “nutritional immunity”, a term coined in the mid-70s [16]. The first hint to this defense strategy’s existence occurred in the mid-40s, when the high-affinity iron-binding transferrin was discovered [17,18]. Immediately after the invasion of infecting bacteria, the body responds, reducing free iron levels in the blood and tissue (hypoferremic response) [19,20]. Posterior studies have shown that nutritional immunity is a strategy that is not limited to iron sequestration and also includes restricting the availability of other essential elements, including zinc [21,22,23,24,25]. In the face of these nutritional limitations, microorganisms evolved stratagems to scavenge sufficient quantities of trace elements necessary to support their metabolism and growth. Zinc is a component of nutritional immunity; in human serum, which contains 0.1% of the total body zinc, about 98% is bound to proteins, mainly albumin (80–85%) and alpha-2-macroglobulin (5–15%), and in marginal quantities to other proteins [26,27,28]. Additionally, zinc is further restricted to pathogens in ongoing infections by releasing calprotectin, a protein that sequesters this metal and creates zinc-limited microenvironments [29,30,31,32]. Calprotectin, a heterodimer formed by the S100A8 and S100A9 proteins, also binds manganese and iron [31,33,34], and it has a proven effect against infection [23,33,34,35,36,37,38]. Other proteins, like the S100 family calgranulin C (S100A12) and psoriasin (S100A7), have also been shown to be able to bind zinc and could contribute to nutritional immunity response [39,40]. As zinc is a component of nutritional immunity, bacteria need to sense the intracellular concentrations and put in motion the different mechanisms involved in this element’s homeostasis. Interestingly, while essential to support growth, zinc is also known to inhibit the progress of infectious processes caused by bacteria [41,42] and viruses, including SARS-CoV-2, the causative agent of COVID-19 [43,44,45,46]. Zinc also inhibits SOS-induced antibiotic resistance and horizontal transfer of antibiotic resistance genes in enteric bacteria [47,48]. Another utilization of zinc as defense by the human host is through macrophages, which use it within the phagolysosome to intoxicate invading bacterial cells [49,50,51,52,53]. A common mechanism by which zinc in excess is toxic to bacterial cells is by binding to noncognate proteins [52,54,55,56].

The effect of zinc in the progress of infection was also investigated utilizing zinc-deficient murine models [45,57,58]. Furthermore, zinc-deficient mice were found to be suitable models for more general studies of infections caused by enterotoxigenic *Escherichia coli*, *Shigella flexneri*, and *Campylobacter jejuni* and potential treatments and immunization [45,57,58].

While zinc uptake is an essential process for bacterial pathogens to cause disease, this element can also be detrimental to some infections. Since long ago, zinc has been used as treatment and prophylaxis of diarrheal diseases [59,60,61]. It was originally thought that the beneficial effect of zinc in treating enteropathogenic *E. coli* (EPEC)-produced diarrhea was entirely caused by enhancement of the immune response and inhibition of ecto-5’-nucleotidase, an enzyme that catalyzes the conversion of the 5’-AMP to the secretagogue adenosine [57,58]. However, this initial idea proved to be insufficient to explain the therapeutic effects observed [57,58,62]. Addition of zinc acetate at sublethal concentrations caused a decrease in the expression of various virulence factors [62]. The mRNA species corresponding to the *bfp* gene (bindle forming protein) and various *esp* genes (EPEC-secreted proteins) were expressed at reduced levels. Furthermore, zinc acetate lowered the bacterial cells’ adherence, inhibited secretion of infection-induced fluids into ileal loops, and reduced histopathological damage in an animal model of infection [62]. Zinc acetate also had effects on the virulence of Shiga-toxigenic *E. coli* (STEC) and enteroaggregative *E. coli* (EAEC) [44,45,63]. It inhibited STEC adherence to cultured cells, expression of enterohemorrhagic *E. coli* (EHEC)-secreted protein A (EspA) and Shiga toxin. In vivo, it reduced fluid secretion and toxin levels in the loops and reduced STEC-induced histological damage [64]. In several forms like oxide, sulfate, and acetate, zinc was also used to test its effects on EAEC [42]. A decrease was observed in biofilm formation, cell adhesion, and expression of other potential or confirmed virulence factors [42]. The observation that zinc reduced the expression of *recA* suggests that inhibition of the SOS response may be one mechanism by which zinc acts on *E. coli* virulence [41]. This finding also prompted other studies to test if zinc could also reduce SOS-induced hypermutation response to antibiotics or horizontal transfer of resistance traits [48,65,66,67]. Zinc blocked the SOS-induced (hypermutation response) development of resistance in *E. coli, Klebsiella pneumoniae*, and *Enterobacter cloacae,* probably by inhibiting RecA binding to single-stranded DNA [47]. Zinc also interfered with horizontal transfer of a β-lactamase gene from *Enterobacter* to *E. coli* strain [47]. As observed with other zinc effects [63,68], the complex zinc ionophore showed significantly higher activity than zinc salts in inhibition antibiotic-induced hypermutation [47].

Environmental enteropathy, a small intestinal disorder caused by subclinical intestinal infections, produces chronic low-grade intestinal inflammation and dysregulation of tight junctions [69,70]. A study on adults with environmental enteropathy showed disruptions that cause leakage in the patients’ epithelial barrier [69]. The authors hypothesized that the sites with epithelial defects could be responsible for bacterial translocation. In vitro experiments utilizing enteropathogenic *E. coli* and *Citrobacter rodentium* showed that these bacteria induced barrier dysfunction, and treatment with numerous compounds like zinc, epidermal growth factor, colostrum, trefoil factor 3, resistin-like molecule-β, hydrocortisone, and ML7 (an inhibitor of the myosin light chain kinase) increased transepithelial resistance while reducing bacterial translocation [71]. This effect was nutrient-independent, and, of all the tested compounds, only zinc exhibited an antimicrobial activity [71].

### 2.2. Zinc Oxide Nanoparticles as Antibacterial

Zinc ions at concentrations higher than those needed for the cells’ normal physiology are detrimental [12]. Multiple effects of excess zinc concentrations lead to bacterial cell death [12,52]. Interestingly, zinc ions at sublethal concentrations inhibit biofilm formation but do not disrupt preformed biofilms in *Actinobacillus pleuropneumoniae*, *Salmonella typhimurium*, *Haemophilus parasuis*, and at a lesser level, *E. coli*, *Staphylococcus aureus,* and *Streptococcus suis* [72]. Zinc oxide is the most common, but not the only, zinc compound used as an antibacterial [73,74], and it has attracted great interest in nanoparticle form [75,76]. Zinc oxide nanoparticles are being researched for applications not only against infections but also as drug delivery tools and as therapies for a variety of conditions [75]. The enthusiasm for nanoparticles’ uses as antibacterial agents is partly fueled by their particular mechanisms of action that differ from those utilized by currently used antibiotics and have targets reducing the frequency of appearance of resistant strains [77,78]. Various research teams have tested the activity of zinc oxide nanoparticles as antibacterials against numerous species [75,76,79,80,81,82,83,84]. The mechanism by which zinc is toxic to bacterial cells is ultimately that of other forms of the ion; however, zinc oxide seems to be more effective when it is administered in nanoparticle form [80,81,85]. Zinc oxide nanoparticles release zinc in an aqueous medium, which then penetrates the cells and produces toxic effects [75]. Zinc oxide nanoparticles are robust candidates to be developed as standalone antimicrobials or as components in combination therapies against multiresistant bacterial infections.

### 2.3. A Brief Overview of Zinc Homeostasis in Bacterial Pathogens

The intracellular concentration of zinc must be tightly controlled to ensure that it is high enough to sustain life but low enough not to cause toxic effects that can lead to cell death [6,12,85,86,87,88]. Through the molecular interactions with biomolecules, zinc participates in a wide variety of metabolic processes as well as repair and maintenance of cell structures and biomolecules that are essential for bacterial cell growth [7,13,81,89,90]. However, higher than needed concentrations of zinc are highly detrimental to the life of the bacterial cell. Infecting bacteria may need to overcome zinc deprivation or toxicity depending on the location of the infection. While nutritional immunity produces deprivation of the metal ions, intracellular and other niches have the opposite effect [50,56]. As a consequence of the strict requirements for intracellular zinc concentrations, bacteria evolved several tightly regulated uptake, efflux, binding, and sensing mechanisms critical for pathogenicity [6,12,23,24,25,33,34,35,36,37,76,86,89,90,91,92,93,94]. This section will discuss the major and better-known mechanisms of zinc import and export. Major regulation mechanisms will be described in the following section.

Import systems can be specific, usually coupled to an energy source, and nonspecific, mostly exerting their function through diffusion gradients across the cytoplasmic membrane [12]. A major import mechanism is ZnuABC, an ATP-binding cassette (ABC) transporter [95,96]. The ZnuA and ZnuB are the periplasmic and inner membrane components, respectively, and ZnuC is the cytosolic ATPase. ZnuA, the periplasmic component, belongs to the cluster A-I of substrate binding proteins [97]. These proteins are associated with ABC transporters in prokaryotes. They are periplasmic in Gram-negatives or lipid-anchored in Gram-positives. They can also exist as domains of periplasmic or inner membrane components [97,98]. The *znuA* gene is transcribed in opposite orientation to *znuC–znuB* [96,99]. The transport complex consists of one molecule of the periplasmic ZnuA and two ZnuC and ZnuB monomers [96,99]. Another component of this zinc uptake mechanism is ZinT (also known as YodA) [100,101,102]. This periplasmic metal-binding protein recruits zinc and delivers the ion to the ZnuABC complex through specific interaction with ZnuA [103,104,105]. It is worth mentioning that ZinT is not exclusively a zinc-binding protein. It shows affinity for other divalent metal ions and also participates in detoxification [106]. In Gram-negatives, there is a need for one or more outer membrane receptors. The protein ZnuD found in bacteria belonging to several families like *Neisseriaceae*, *Moraxellaceae, Acinetobacteriaceae*, *Pseudomonadaceae,* and *Bordetellaceae* was proven to be a zinc transporter and a requirement for a high virulence phenotype [107,108]. The 82 kDa ZnuD is a member of the TonB-dependent siderophore receptor family of proteins, and it may be able to recognize free or metallophore-complexed zinc ions [107]. ZnuD has been identified as a potential candidate for a vaccine against at least two bacteria: *Acinetobacter baumannii* and *Neisseria meningitidis* [103,109]. Interestingly, while the *znuD* gene is included in the *A. baumannii* chromosome, another homolog, *znuD2*, is present in about half of the isolates, in at least one case within a plasmid [93]. The role of ZnuD in *A. baumannii* virulence was proven but that of ZnuD2 remains to be clarified because of difficulties in generating a mutant that inactivates the gene [93]. Although most studies about the ABC transporter mechanism of uptake of zinc were carried out on Gram-negative models, research on Gram-positives showed that this mechanism also exists in these bacteria. The proteins AdcB and AdcC are the integral membrane and ATPase components, respectively. In addition, there are substrate-binding lipoproteins, AdcA and AdcAII, that are attached to the membrane’s surface and are the functional equivalents to ZnuA and ZnuT [110]. Structural and functional studies showed that AdcA and AdcAII are substrate-binding proteins that bind zinc, but they do it via different structural features [97,110]. In particular, the structure of AdcA resembles a fusion between the zinc-binding domains of ZnuA and ZnuT [110]. Although AdcA and AdcAII have redundant functionalities in vitro, they are both required for full virulence in *Streptococcus pneumoniae* [110].

An outer membrane protein found in *N. meningitidis* is CbpA, which binds calprotectin in complex with zinc ions and, in a process that resembles the uptake of iron from transferrin or lactoferrin, mediates acquisition of zinc in a TonB-dependent manner [104].

Until recently, the mechanism to scavenge an essential metal ion by means of small molecular weight, high-affinity compounds known as siderophores was mostly known for the uptake of ferric iron. Siderophores compete with the host organism’s high-affinity binding proteins, and the ferric–siderophore complexes are internalized into bacterial cells through specific receptors [105,111]. Recent research showed that bacteria possess a zinc uptake mechanism that mimics siderophore-mediated iron-uptake systems. Metal chelators, which are chemically unrelated to siderophores but play a similar role transporting several metals, were found in many bacteria [112]. They are known as metallophores, and when they transport zinc, are sometimes called zincophores. A broad-spectrum metallophore, staphylopine, produced by *S. aureus*, is one of the earliest metallophores found in a human pathogen [112]. Staphylopine participates in the transport of nickel, cobalt, zinc, copper, and iron [112]. Furthermore, the staphylopine biosynthetic pathway is conserved in numerous bacterial pathogens, and in some cases, it is associated with virulence [112,113]. Although other zincophores were subsequently discovered, research on these elements is in its infancy. Many more will most probably be detected and characterized in the future [90,114,115,116].

ZIP (*z*inc–iron permeases or ZRT/IRT-like proteins) mediate zinc uptake into the cell’s cytosol. They were thought not to exist only in bacteria, but it is now known that they can be found in all kingdoms [117]. Expression of some of them is regulated by zinc concentration like the Zip11 and Zip63 from *Nostoc punctiforme* [118] or the *Salmonella enterica* ZupT, which was shown to be a virulence factor [119]. Conversely, expression of the *E. coli* ZupT seems to be constitutive [120].

Transcriptomic analyses using mutants that lack a functional Zur (*z*inc uptake regulator, a regulator discussed below) led to the identification of elements involved in zinc homeostasis [99,121,122]. One of them, the *A. baumannii* ZrlA, is a zinc-binding peptidase induced in zinc-limiting conditions. A lipoprotein anchored to the inner membrane, ZrlA is necessary for appropriate uptake levels under zinc starvation and may promote uptake through modifications of the peptidoglycan [94]. Interestingly, mutants lacking a functional ZrlA are also more susceptible to antibiotics. Both effects may be related to the protein’s contribution to cell wall integrity [94]. Another protein involved in *A. baumannii* zinc homeostasis is ZigA, a zinc-binding GTPase necessary for full growth in zinc-limiting conditions and full virulence [123].

Since zinc is highly toxic at higher concentrations, bacteria are also equipped with efficient efflux systems that contribute to the homeostasis. Gram-negative as well as Gram-positive bacteria can export zinc through P-type ATPase transporters, a ubiquitous superfamily of membrane ion transporters that couple ATP hydrolysis to ion passage from inside to outside of the cell [97,124,125]. The presence of P-type ATPase transporters in cells from all kingdoms of life and the characterization of many of them led to their classification in at least five subfamilies, P1–P5, that are further subdivided (P1B–P1B, P2A–P2D, P3A–P3B, and P5A–P5B) [126]. Zinc is mostly exported through P1B-type ATPase transporters, which can also transport other metal ions [91,92]. The ATPase’s general structure includes a transmembrane domain that has a central core of six α-helices and a cytoplasmic portion that contains three domains: nucleotide-binding, phosphorylation, and actuator. The protein adopts two possible conformations, E1 and E2, and each one can be phosphorylated (E1P and E2P) or not. In conformation E1, the metal ion can reach an intramembrane site, and ATP–Mg can bind the nucleotide-binding site. The protein adopts the E1P state after phosphorylation by the transfer of a phosphate from ATP. A conformational change to E2P closes the possibility that the metal ion flows back to the cytosol. A further conformational modification releases the metal ion to the outside of the cell, followed by hydrolysis of the phosphate group, which leads to the adoption of the E2 state [92,126]. Several P1B-type ATPase transporters from Gram-negative and Gram-positive bacteria have been mechanistically and structurally studied, and many of them were confirmed as virulence factors [127,128,129,130,131,132].

Another family of transporters that export zinc outside the cytosol is the cation diffusion facilitators (CDF). Proteins in this family are subdivided into groups according to the metal they transport [121,122,124]. In prokaryotes, these transporters play a role in metal ions homeostasis, tolerance, and resistance [121,122,125,133,134]. ZitB, an *E. coli* member of the CDF family, was first shown to be an antiporter that effluxes the divalent metal cation in exchange for protons [135]. Studies on CDFs in several bacteria later confirmed that they export the cations through an antiport mechanism driven by the proton motive force [136,137,138]. In at least one case, the exchange could also occur with potassium [139].

The structure of the 32 kDa CDF YiiP protein from *E. coli* has been resolved. It exists as an integral membrane Y-shaped homodimer with a C-terminal 89-amino acids cytoplasmic domain [140,141]. The two upper arms of the Y are the transmembrane domains, which include six α-helices, and the lower stalk of the Y is the C-terminus cytoplasmic region. Studies on YiiP and other structures that were resolved indicate that the C-terminal region is structurally related to metallochaperones. Therefore, one of the functions of this sector of the protein could be to sense zinc and deliver it to the transmembrane region [137,140,141,142,143,144]. At least two mechanisms have been proposed for the transfer of zinc through the cytoplasmic membrane. Both consider conformational changes as the ion contacts the protein, and it is subsequently exported [121]. Interestingly, CDF proteins that lack the C-terminus have been recently identified. Although the existing evidence points to a role in the export of zinc and other metal ions, research of these elements is still in the early stages [121,145].

The CDF and ATPase-mediated export mechanisms of Gram-negative bacteria deliver zinc to the periplasmic space, and then the ions must be exported outside of the cell wall. Conversely, heavy metal efflux (HME) systems, which belong to the resistance–nodulation–cell division efflux system superfamily, transport zinc from the cytosol to the milieu [13,95,97,146]. Three proteins assemble the HME export machinery. Components known as A and C are integral inner and outer membrane proteins, respectively. They usually exist as trimers and are connected through a periplasmic protein component (B) that forms a hexameric or trimeric ring around the inner and outer membrane components [147]. A HME system from *A. baumannii*, *czcCBA*, was recently studied, and it was found that all three genes are highly regulated, together with the CDF proteins coded for by *czcE* and *czcF*, when the cells were treated with 2.5 mM zinc sulfate for 1 h [148]. This work found that cytoplasmic zinc can be transported from the cytoplasm to the periplasmic space by the CDF member CzcD; a metallochaperone CzcI may bind the metal ion and deliver it to the CzcCBA system that completes the efflux process [148].

More detailed descriptions of bacterial systems and elements involved in zinc homeostasis can be found in numerous excellent reviews and publications [12,36,56,85,86,91,92,119,121,122,123,124,126,131,132,147,149,150].

### 2.4. Zinc-Driven Regulation of Gene Expression

Cells keep a delicate balance to avoid zinc deficiency or toxicity. For this, they are equipped with numerous systems, some of them discussed in the previous section, to take up or export zinc. Cells possess regulators, mainly transcription factors, that control the levels of expression of the different uptake and efflux systems to ensure that ions are imported or exported according to their needs [6].

The Fur family is an essential group of metal sensors that regulate gene expression. The first known member of this group of proteins was Fur (ferric uptake regulator), which functions as a classical repressor, interfering with transcription when complexed to iron [151,152]. Further research showed that most bacteria possess Fur homologs [15,146,153,154,155,156,157,158]. Fur is a dimer that binds iron, which acts as corepressor and activates it to favor binding to the Fur box (an operator) to turn off the expression of genes mainly involved in iron-uptake systems [159,160]. While this is the only identified factor regulating gene expression in many cases, Fur can serve as part of regulatory circuits that inhibit expression of genes when the concentration of cellular iron is high [161,162]. Fur also acts as a positive regulator of certain genes by repressing expression of a protein required for expression of another gene, facilitating binding of RNA polymerase when binding to the Fur box located upstream of a responsive gene, or binding to a Fur box located near an operator and interfering with repressor binding to such an operator [163]. Fur functions as a dimer and binds one iron per monomer. It binds iron at the C-terminus region and the DNA Fur box through a winged-helix DNA-binding domain located at the N-terminus [164]. A signature, histidine-rich motif (HHHXHX_2_CX_2_C), is located close to the dimerization domain between the DNA-binding domain and the C-terminal region [153,164]. Research on Fur through the years have shown that related regulator proteins have different metal-binding sites with affinity for other metal ions or compounds, such as hydrogen peroxide. Of particular interest to this article are the Zur regulators, which, mirroring the Fur proteins, act mainly, but not always, as negative regulators of gene transcription and repressors of uptake of zinc [6,165]. A search at the Research Collaboratory for Structural Bioinformatics Protein Data Bank revealed that three crystal structures of bacterial Zur proteins have been resolved [166,167,168]. The *E. coli* Zur structure was solved in association with zinc and bound to the promoter region of the *znuABC* operon [166]. The authors showed repression of gene expression mediated by highly cooperative binding of two adjacent dimers [166]. Studies using other Zur-regulated promoters showed significant differences in binding affinity between Zur–zinc complexes and the cognate DNA protein binding sites (Zur boxes) and the strength of inhibition of gene expression [166]. Zur proteins, as it is the case of other Fur family members, have two conformations. When bound to zinc (activated, closed conformation), it has high affinity for the Zur binding DNA regions. When not complexed to the metal (open conformation), it has negligible affinity for DNA [6]. Numerous experiments designed to elucidate aspects of regulation of gene expression by Zur and its requirement for virulence have shown that most of the time, albeit not always, it is essential for virulence [169,170]. An extensive listing of bacterial operons regulated by Zur can be found in a recent review [6].

A zinc-dependent gene regulator with functions similar to Zur, but with unrelated structural characteristics, has been identified and characterized in different species of *Streptococcus* [56,171,172,173]. This regulator, AdcR (adhesion competence resistance), is part of the MarR family of transcription factors. Proteins in this family participate in the regulation of expression of genes involved in virulence, antimicrobial resistance, and other physiological functions [174,175]. Similarly to Zur and other metalloregulators, AdcR is a homodimer that includes a DNA-binding and a zinc-binding domain. After associating with zinc, a conformational change increases the affinity for DNA operator sequences (known as *adc* motif) and leads to binding and inhibition of gene expression [176]. A recent study on *Streptococcus pyogenes* showed that by sensing the cytosol’s zinc concentration, AdcR regulates about 70 genes. As is the case with other metalloregulators, AdcR acts as a negative and positive regulator, and its effects extend to functions other than zinc homeostasis, including virulence and metabolism [171].

**Figure 1:**
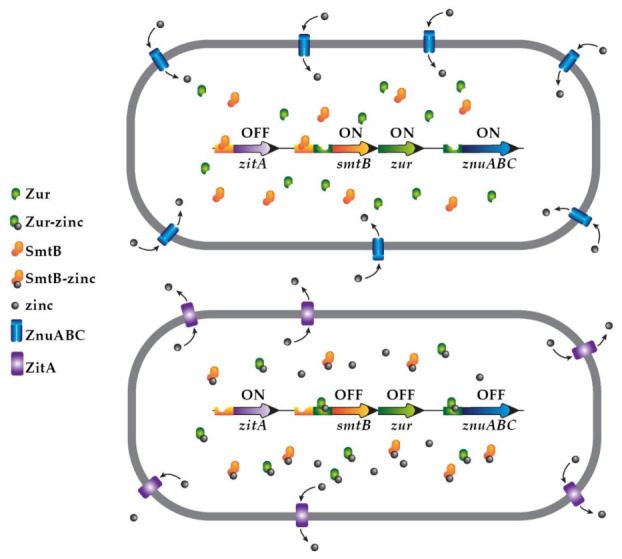
*Mycobacterium smegmatis* SmtB and Zur regulation of gene expression in response to the concentration of zinc. Model proposed by Goethe et al. [185]. The upper diagram shows a cell in an environment with low zinc concentration. In these conditions, SmtB is a homodimer that acts as a repressor of the expression of ZitA (zinc exporter). Binding of ZitA also facilitates expression of the *smtB* and *zur* genes. Zur depleted from zinc is unable to efficiently bind the Zur boxes upstream of *znuABC* (zinc importer) and *smtB*/*zur*. As a consequence, expression of ZnuABC and the regulators set up the cell to internalize zinc ions. The bottom diagram shows a condition where zinc ions are abundant; Zur binds the metal ions as a homodimer and binds the operators, turning off expression of *smtB*/*zur* and *znuABC*. SmtB loses affinity to the DNA, permitting high expression of ZitA, which facilitates export of zinc ions.

Mechanisms to export zinc outside the bacterial cell, discussed in a previous section, are necessary to counter the host immune system based on zinc intoxication. They were first discovered when studying interactions between macrophages and *Mycobacterium tuberculosis* [49,53] and later described as a host defense against some enterobacteria and *Streptococcus* [50,51]. As is the case for systems that mediate uptake, expression of export systems is tightly regulated. The expression of the exporter CzcD proteins of *S. pyogenes*, *Streptococcus agalactiae,* and *S. pneumoniae* are activated by GczA (*S. pyogenes* and *S. agalactiae*) or SczA [50,177]. These are related transcriptional regulators that belong to the TetR family [50]. As is the case for the regulators Zur and AdcR, these regulators can also have the opposite function to which they were discovered. Therefore, GczA and SczA can also act as a negative regulator [177,178]. A recent study on SczA showed that it is a homodimer, with each monomer containing two zinc-binding sites necessary for transcriptional activation [177]. Studies on different species of *Mycobacterium* showed that as opposed to Zur’s action, the SmtB binds DNA when it is bound to zinc, which happens when the metal ion concentration is too high. In consequence, an increase in the concentration of zinc results in detachment of SmtB from the operator region, inducing expression of zinc exporters like ZitA [179,180,181]. It is of interest that SmtB and Zur are part of an operon in *Mycobacterium* species, a characteristic that seems to be specific to mycobacteria and a small group of actinobacteria and *Corynebacterium diphtheriae* [1,182,183,184]. Goethe et al. proposed an elegant model of regulation of gene expression in *Mycobacterium smegmatis* at high and low zinc concentrations mediated by Zur and SmtB. Both regulators are expressed from a unique operon containing binding sites for both proteins (Figure 1) [185]. At low zinc concentrations, apo-SmtB binds the DNA and induces expression of the regulators (apo-SmtB and apo-Zur), then apo-SmtB binds the operator of the gene coding for an exporter protein [185]. Meanwhile, the expression of importers is allowed to proceed because apo-Zur does not bind DNA efficiently. At high zinc concentrations, the holo-Zur binds the Zur binding site upstream of the *smtB–zur* operon and represses the expression of SmtB. It also binds the operator regions of genes coding for zinc importers. SmtB bound to zinc does not bind DNA, and zinc exporters are therefore expressed (see Figure 1) [185].

### 2.5. Zinc as Adjuvant to Antibiotic Treatment

Aminoglycoside antibiotics have a general chemical structure consisting of an aminocyclitol nucleus (streptamine, 2-deoxystreptamine, or streptidine) linked to amino sugars [186,187,188]. They are used to treat a broad spectrum of bacterial infections. Aminoglycoside modifying enzymes are responsible for the vast majority of aminoglycoside treatment failure in clinics [182,189]. Because therapies combining an antibiotic and an inhibitor of resistance proved to be potent weapons against β-lactam-resistant infections [190,191], there have been numerous attempts to find equivalent compounds to overcome resistance to aminoglycosides caused by modifying enzymes [183,184,190,192,193,194,195].

Recent studies to identify inhibitors of this resistance mechanism showed that zinc interferes with enzymatic inactivation by acetylation catalyzed by the aminoglycoside 6’-*N*-acetyltransferase type Ib (AAC(6’)-Ib) and others [68,193,196,197,198]. The initial experiments showed that zinc ions very efficiently inhibited AAC(6’)-Ib-mediated acetylation of aminoglycosides in vitro [68]. However, attempts to interfere with resistance in cellulo by treating bacteria with amikacin and zinc chloride (ZnCl_2_) required enormous concentrations of the salt. The differences between the concentrations necessary for efficient inhibition in vitro and in cellulo could be due to permeability issues or the action of zinc export systems (see previous sections). Therefore, the combination treatment was modified to include ionophores that could increase the number of zinc ions that reach the cytosol. The addition of zinc complexed to pyrithione reduced the concentration required to add to the growth medium to reverse resistance to amikacin by 1000-fold [68,196]. Similar results were obtained utilizing 8-hydroxyquinoline analogs, such as clioquinol or 5,7-diiodo-8-hydroxyquinoline [63,197]. An aminoglycoside-resistant *A. baumannii* strain that resists these antibiotics’ effects through a mechanism other than enzymatic modification grew equally in the presence of amikacin or amikacin plus clioquinol or 5,7-diiodo-8-hydroxyquinoline ionophores [63]. These experiments expanded the scope of zinc’s therapeutic uses to encompass being an inhibitor of the clinically most relevant resistance mechanism to an important group of antibiotics as it is of aminoglycosides.

## 3. Eukaryotes

### 3.1. Importance of Zinc in Eukaryotic Cells

The effectiveness of antibiotics against bacteria exemplifies the importance of these drugs against many human infections. Unfortunately, the same drugs that kill bacteria have similar effects on the mitochondria present within eukaryotic cells [199]. The synergistic effect of zinc with antibiotics may also be the reason why zinc itself is toxic to eukaryotic cells at high intracellular levels because zinc enters the mitochondria and enhances production of reactive oxygen species (ROS) [200,201].

In eukaryotic cells, zinc exists in labile or free ion form (chelatable) [202], while zinc bound to proteins serve in a structural or catalytic capacity [202,203,204]. Zinc is a small ion (~0.65 angstrom) that binds nitrogen- and sulfur-containing molecules and readily exchanges ligands due to its low ligand field stabilization energy. Intracellular zinc levels range from 10^−12^ to 10^−9^ M in most cells, but zinc-enriched cells such as neurons, hepatocytes, splenocytes, and thymocytes may contain an estimated 10^−6^ to 10^−5^ M amount [205,206,207,208]. Zinc concentrations range from 10^−9^ M within the cytoplasm in most cells to 10^−3^ M in some vesicles [209]. In transgenic baby hamster kidney cells that express zinc influx transporter proteins, Palmiter and colleagues (1996) estimated that vesicular zinc concentrations reach 14 µM when these cells are exposed to high levels of exogenous zinc [210]. Cell survival in vitro is compromised when cells are exposed to extracellular zinc concentrations ([Zn^2+^]e) between 225–1000 µM in neuronal cells and 7.5–200 µM in non-neuronal cells [211,212,213,214]. It is therefore essential that cells regulate their intracellular zinc concentrations through protein influxers and effluxers as well as physiological chelation by apo-thionein or other zinc-sequestering apoproteins [210,215,216,217,218].

### 3.2. Zinc-Rich Cells

Zinc-rich cells in mammals are found in various tissue and organs, particularly in the brain, mammary gland, intestine, pancreas, thymus, prostate gland, testes, and ovaries [202,219,220,221]. In the brain, high levels of chelatable or labile zinc is synaptically co-released with glutamate during normal neuronal communication [202], with ionic levels reaching 100–300 µM, particularly during a seizure activity [222,223]. Intracellular zinc is typically buffered but is exocytosed from neurons or secretory cells that release vesicles or granules, respectively. Zinc release in the hippocampal mossy fiber terminals is calcium-dependent whether it was evoked via potassium or kainic acid administration and spontaneous activity [222,224,225,226]. Intracellular zinc elevation may occur due to high exogenous zinc levels [227,228,229,230,231] or caused by cytoplasmic zinc release from compartments or proteins due to oxidation by ROS or nitrosylation by reactive nitrogen species [232,233,234]. Zinc overload kills neurons, and thus it is imperative that cells tightly regulate intracellular zinc concentration via zinc transporters and buffering of zinc-binding amino acids or proteins.

### 3.3. Zinc Transporters

High- and low-affinity uptake mechanisms for zinc have been identified with dissociation constants (Kd) of 15 and 361 µM, respectively [222]. An even higher binding affinity constant (Ka) of 0.25 µM has been reported for zinc, which is saturable, ATP-independent, and unaffected by Na^+^ concentration gradient [235]. Indeed, zinc levels are strictly maintained by tissue-specific and highly conserved low molecular weight transport protein families known as the ZnTs (also known as SLC30 for solute carrier 30) and ZIPs (also known as SLC39 for solute carrier 39) [236,237,238]. Early studies in the field led to the discovery of mammalian ZnTs involved in the extrusion of zinc out of cells named ZnT1 [215] and sequestration of zinc into compartments called ZnT2 [210]. ZnT1 is mainly localized in the plasma membrane [215]. Meanwhile, ZnT2 is localized within vesicular (acidic) compartments, such as lysosomes, but has a low affinity for zinc [210]. Another effluxer termed ZnT3 was cloned and identified to localize within synaptic vesicles of zinc-rich neurons [217]. ZnT3 is expressed in the mammalian brain, such as in the cerebral cortex and the hippocampus, and strongly detected in the dentate granule cells. Over the course of time, other members of the ZnT effluxers (ZnT4–ZnT10) and ZIP influxers (ZIP1–ZIP14) were identified through sequence similarity analyses, cloning, and functional experimentations [223,238]. More recently, transmembrane 163 protein (TMEM163; also known as SV31) [239,240] was functionally characterized as a dimeric protein that effluxes zinc [241], and one of us proposed that TMEM163 be now classified as ZnT11 as a new member of the ZnT efflux family of proteins [241]. One commonality among ZIPs and ZnTs is that histidine (H) and/or aspartic acid (D) residues, such as the HXXXD motif (where X is a nonpolar amino acid) typically located within transmembrane domain (TMD)-4 and TMD5 helices of ZIPs, as well as HXXXH motif found in TMD2 and TM5 helices of ZnTs have been shown to be responsible for tetrahedral zinc coordination [223,242]. For a relevant review on certain zinc transporters, we refer the reader to the paper by Styrpejko and Cuajungco (2021) as part of this Special Issue.

In addition to transporters, intracellular buffers offer a secondary defense mechanism to prevent intracellular zinc overload, such as the metallothioneins (MTs)—a group of low molecular weight (~6–7 kDa), single polypeptide chains with four functional mammalian isoforms (MT1−MT4) [219,243]. MTs, however, are not a long-term storage for zinc due to its short biological half-life [244]. Thus, vesicular or compartmental storage mediated by ZnTs and ZIPs provide important contributions to zinc homeostasis.

### 3.4. Zinc Interactions with Various Proteins

Zinc is crucial to over 200 proteins and enzymes [204]. Most of the endogenous and exogenous zinc in biological systems is bound to proteins with different degrees of affinity (see Table 1). The ultrastructural localization of several neurotransmitters present in the hippocampal mossy fiber terminals, especially glutamate, coincides with the location of zinc-containing neurons [245,246,247,248]. Zinc is known to bind and permeate ionotropic glutamate receptors (e.g., amino-3-hydroxy-5-methyl-4-isoxazole propionic acid (AMPA), kainate, and N-methyl-D-aspartate (NMDA)) and metabotropic glutamate receptors [249,250,251,252,253]. Zinc also binds to the gamma-amino-n-butyric acid (GABA) receptors and noncompetitively inhibits GABA-mediated responses [254,255]. Zinc antagonizes both NMDA [256] and GABA [257] with approximate Kd of 13 and 11 μM, respectively. These are well within the concentrations released during synaptic activity, which implies an association of zinc in the regulation of neurotransmission. Likewise, zinc can modulate the responses of ligand-gated or voltage-gated ion channels, such as the ATP-gated P2X-purinergic ion channels, glycine, and sigma receptor [258,259,260].

#### 3.4.1. Enzymes

Zinc impedes the activity of several ionic transporters, such as the sodium–potassium (Na^+^/K^+^)-ATPase with IC50 = 20 µM [262,275] and Ca^2+^-ATPase in vitro IC50 = 100 µM (Table 1) [276]. The Na^+^/K^+^-ATPase enzyme activity has been found to be highly concentrated in the hippocampus and hypothalamus [275], and the inhibition of Na^+^/K^+^-ATPase has been shown to cause neuronal death [277,278].

Zinc can inhibit the cell respiratory chain (Ki = 10^−7^ M; see Table 1) by blocking the initial step of respiration of electron transfer between ubiquinone (coenzyme Q) and cytochrome b of the bc1 complex (complex III) [264,279]. At higher concentrations (10^−3^ M), zinc may further inhibit at the levels of flavoprotein 1–2 (complex I–II) and cytochrome c oxidase (complex IV) activities [280]. The inhibitory effects of zinc may explain why mitochondrial zinc overload is cytotoxic to eukaryotes.

In addition to inhibitory effects on energy metabolism, zinc also negatively impacts a number of enzymes critical for neurotransmitter metabolism [202]. For example, it was reported that zinc inhibits glutamate and GABA reuptake transporters (Km ~50 μM) in mouse synaptosomal fractions [281]. Although zinc inhibition of glutamate transporters results in extracellular glutamate accumulation and death of cortical neurons in vitro [282], it was reported that physiological levels of zinc released within the synapse facilitate glutamate homeostasis through its effect on glutamate transporter activity [283]. For further information regarding the inhibitory effects of zinc on additional enzymes, see the review by Maret (2013) [284].

#### 3.4.2. Signaling Proteins

It has been observed that zinc influx may depolarize the cell and consequently trigger a rise in intracellular calcium concentration ([Ca^2+^]i). Calcium is then bound by a number of EF-hand proteins that contain distinct zinc binding sites, such as S-100A6 (calcyclin), S-100A7, S-100A8, S-100A9, S-100A12, calmodulin (CaM), and calgranulin C (CAGC) [272,285,286]. Zinc binds CaM (Kd ~8 × 10^−5^ M) and induces a slight conformational change; however, calcium has a greater affinity for CaM (Kd = 10^−8^ M) [272]. The binding of zinc to these calcium-binding proteins under physiological conditions remains a point of contention. Notwithstanding, recent reports indicate that zinc and calcium play important roles in the function of these S100 proteins [287].

#### 3.4.3. Cytoskeletal Proteins

Zinc has been shown to influence both assembly and disassembly of tubulin (Table 1) and microtubule-associated proteins in vitro [273,288]. An excess of zinc levels in nervous tissues could interfere with the microtubule structure by disrupting the normal functions of the cytoskeleton [289]. The metal chelator, *N,N,N,N*-tetrakis(2-pyridylmethyl)ethylenediamine (TPEN), prevented protein kinase C (PKC)-mediated actin cytoskeletal disruption induced by phorbol ester in cultured C6 rat glioma cells, while addition of zinc reversed the protective effects of TPEN [290].

### 3.5. Zinc-Induced Cell Death

Abnormal zinc metabolism results in an excess or deficiency of intracellular zinc concentrations ([Zn^2+^]i), which could be detrimental to cells. The mechanism of zinc-induced cell death has been extensively researched. Zinc may augment or directly trigger cell death through its effects on various cellular pathways. The following discussion summarizes the connection between zinc and programmed cell death (apoptosis).

#### Zinc Mobilization in Apoptosis

Apoptosis is characterized by distinct morphological changes in cells that include membrane blebbing, chromatin condensation, DNA fragmentation, organellar packaging, and cell shrinkage [291]. Cells undergoing apoptosis induce zinc-dependent transcription factors [292]. Recently, cell death involving iron has been observed and coined ferroptosis (for reviews on this topic, see the following references [293,294]).

Cultured lymphoblasts undergoing early events of apoptosis exhibit an increase of [Zn^2+^]i [208,295]. Mobilization of [Zn^2+^]i is possibly due to compartmental release and zinc dissociation from metalloproteins mediated by free radicals and ROS such as HOCl, H_2_O_2_, and O^2−^ [296,297,298]; amino acids such as L-glutamate, L-aspartate, and L-cysteic acid [299]; and disulfides [271]. It remains to be shown, however, if intracellular zinc mobilization is a primary or secondary cause of apoptosis. As a case in point, apoptosis is observed in animal models of a neurological disorder called mucolipidosis type IV (MLIV), which is caused by the functional loss of the TRPML1 ion channel [300,301]. Coincidentally, lysosomal permeabilization and the release of cathepsin B have been shown in a cell culture model of MLIV [300], which correlates with lysosomal zinc accumulation in MLIV cells, as well as cell culture and mouse models of MLIV [237,248,302]. It is worth noting that in the MLIV mouse model, the downregulation of the ZnT3 vesicular zinc transporters may explain why cortical zinc levels are abnormal [303], but further research is necessary to determine if this is a cause or consequence. Notwithstanding, targeting [Zn^2+^]i to reduce pathological conditions observed in certain human diseases could be a therapeutic approach. Indeed, in the case of cerebral ischemia [304] or seizure activity [305], degenerating neurons show increased zinc staining, and these neurons undergo an apoptotic process [306]. In the case of ischemia, the pathological process results in [Zn^2+^]i elevation but precedes other biomarkers of cellular damage. Interestingly, neuronal death is rescued by the chelator ethylenediamine tetraacetic acid (EDTA), which suggests that zinc mobilization influences cell death, at least in cerebral ischemia [304].

Intracellular zinc mobilization has biphasic effects on non-neuronal cells by either initiating or preventing apoptosis [221,222,307]. There is, however, a variable range of concentrations where zinc may induce or preclude apoptotic death. For example, cultured mouse thymocytes incubated with zinc at concentrations of 15 μM underwent apoptosis [212], while other studies have found zinc-induced apoptotic death between 80 and 200 μM [213]. These results contrast markedly with induction of *c-myc*-dependent apoptosis by zinc (37.5 µM) in the absence of DNA fragmentation [292]. Based on these findings, intracellular zinc elevation in non-neuronal cells undergoing apoptosis may be a protective mechanism, while zinc mobilization in post-ischemic neuronal cells would appear to be neurotoxic because a metal chelator rescues cell death.

### 3.6. Zinc Chelators

Metal chelators have unique and specific binding properties on various metal ions [308,309]. Certain chelators are able to permeate cell membranes or become lipid soluble, while others become membrane permeable after esterification or by acquiring a nonpolar state following metal complexation. Further, some chelating agents demonstrate particular attributes known as ionophores. Ionophores selectively enhance the permeability of metal ions in lipid membranes of cells [308]. For example, pyrithione and clioquinol are zinc chelators that also act as ionophores [302,310,311,312], in which both compounds increase intracellular zinc by virtue of their membrane permeable property. Meanwhile, a number of chelators avidly associate and sequester metals from metal–protein complexes, while others are incapable of such interaction. The former are classified as high-affinity metal chelators, while the latter are low-affinity chelators. Moreover, metal complexing agents have different denticity or means to bind metal ions. Whereas some chelators form multidentate complexes, others can only bind a mono- or bidentate complex. Note that multidentate ligands do not necessarily result in the formation of high-affinity metal–ligand complex [309]. Thus, properties of chelating agents may depend on the affinity or stability constant they form with the target ion(s), the metal-to-ligand ratio, and the accessibility of the ion within the metal–ligand complex that the chelator is in competition with. Overall, researchers must fully consider the characteristics of the chelator before they use them for experiments.

#### Chelators That Bind Zinc and Their Effects on Cells

Certain chelating agents could exhibit high binding affinity for a particular metal ion; however, these chelators may also bind other metals, albeit less avidly than the target metal. Such a problem of metal selectivity is typically encountered in biological studies. For example, diethylenetriamine pentaacetic acid (DTPA) avidly binds zinc but forms more stable complexes with copper and iron [313], while diethyldithiocarbamate (DEDTC) and zincon bind copper even though both can also complex zinc. Note that DTPA and zincon do not affect histochemical staining for brain zinc, suggesting that both are membrane impermeable, but DEDTC can penetrate cell membrane and quench histochemical stain for brain zinc [314]. One commonly used and highly specific zinc chelator is TPEN. However, although it is highly selective for zinc than calcium or magnesium, it also has a higher affinity for copper and iron [307,315]. One advantage of TPEN is its cell membrane permeability [307,315]. Because TPEN avidly binds zinc, its use to chelate intracellular zinc results in induction of apoptosis in thymocytes [217], lymphocytes [295,316], isolated hepatoma cells [317], and splenocytes [208]. These results are comparable to other reports in which apoptosis was induced by exposure to low levels of zinc [212,213]. Note that zinc chelation-mediated death is not only independent of, but also additive to apoptosis induced by exogenous addition of Ca^2+^ [318]. These observations show that zinc is important for apoptotic cell death pathways.

The use of metal complexing agents against certain disease states is quite common. Indeed, membrane permeable chelators, such as TPEN and pyrithione, attenuate zinc neurotoxicity in vivo [314,319], while the membrane impermeant chelator EDTA reduces zinc toxicity in vitro [211,219,304,314,319,320,321]. It is interesting to note that intrahippocampal co-administration of zinc with several metal chelators have been reported to produce differential effects on neuronal damage in vivo. Specifically, several zinc–chelator complexes showed behavioral side effects, such as seizures, which also correlated with increased neuronal loss [314]. In contrast, however, the same chelators intra-hippocampally injected alone were not significantly toxic compared with the zinc chelate treatment.

In summary, the problem of specificity may be overcome by using metal chelators with varying degrees of affinity for zinc and determining different chemical structures that inform denticity for zinc. Finally, recent advances have now allowed researchers to develop chelating agents with more specificity and affinity for zinc than other metal ions.

### 3.7. Concluding Remarks

Zinc is critical for the growth and survival of cells. However, an abnormal metabolism of zinc in cells can have deleterious effects. Zinc is required by a number of transcription factors, proteins, and enzymes. Studies have shown that zinc can induce cytotoxicity in prokaryotic and eukaryotic cells once a threshold is reached. Zinc inhibits many critical enzymes and regulates receptors or ion channels. Finally, zinc plays a role in apoptotic death and is thus a potential target of specific chelating agents. The importance of zinc in cells cannot be overstated, but further research is necessary to determine when and how zinc may be used for therapeutic intervention in the case of antibiotic resistance as antimicrobial adjuvant and when zinc can be tackled to prevent cell death in various human diseases.

## Figures and Tables

**Table 1:** Tabulated list of selected proteins and enzymes with known binding affinity and inhibitory constant for zinc.

Protein Name	Binding Affinity * [Reference]
**Superoxide dismutase (Cu/Zn)**	Kd = 5 × 10^−5^ M [261]
**Na^+^/K^+^ ATPase**	Kd = 3 × 10^−6^ M [262]
**Ca^2+^ ATPase**	Ki = 8 × 10^−12^ M [263]
**Mitochondrial complex III**	Kd = 1 × 10^−7^ M [264]
**Protein tyrosine phosphatase**	Ki = 2 × 10^−13^ M [265]
**Protein kinase C**	Kd = 1 × 10^−13^ M [266]
**Caspase-3**	Ki = 1 × 10^−13^ M [267]
**Caspase-6**	Ki = 1 × 10^−13^ M [268]
**Sp1**	Kd = 1 × 10^−10^ M [269]
**Glutathione**	Kd = 5 × 10^−7^ M [270]
**Metallothionein**	Kd = 1 × 10^−12^ M [271]
**Calmodulin**	Kd = 8 × 10^−5^ M [272]
**NMDA Receptor**	Kd = 1 × 10^−7^ M [256]
**GABA receptor**	Kd = 1 × 10^−7^ M [257]
**Tubulin**	Kd = 2 × 10^−7^ M [273]
**Heme**	Kd = 2 × 10^−8^ M [206]

* Kd = dissociation constant; Ki = inhibitory constant. Adapted and modified from [274].

## References

[1] Andreini I., Banci C.L., Bertini I., Rosato A. (2006). Zinc through the three domains of life. J. Proteome Res..

[2] Maxfield L., Crane J.S. (2020). Zinc Deficiency.

[3] Andreini C., Bertini I. (2012). A bioinformatics view of zinc enzymes. J. Inorg. Biochem..

[4] Banci L., Bertini I., Ciofi-Baffoni S., Finney L.A., Outten C.E., O’Halloran T.V. (2002). A new zinc-protein coordination site in intracellular metal *Traffic*king: Solution structure of the Apo and Zn(II) forms of ZntA(46–118). J. Mol. Biol..

[5] Debela M., Magdolen V., Grimminger V., Sommerhoff C., Messerschmidt A., Huber R., Friedrich R., Bode W., Goettig P. (2006). Crystal structures of human tissue kallikrein 4: Activity modulation by a specific zinc binding site. J. Mol. Biol..

[6] Mikhaylina A., Ksibe A.Z., Scanlan D.J., Blindauer C.A. (2018). Bacterial zinc uptake regulator proteins and their regulons. Biochem. Soc. Trans..

[7] Coleman J.E. (1992). Zinc proteins: Enzymes, storage proteins, transcription factors, and replication proteins. Annu. Rev. Biochem..

[8] Loh S.N. (2010). The missing Zinc: p53 misfolding and cancer. Metallomics.

[9] Namuswe F., Berg J.M. (2012). Secondary interactions involving zinc-bound ligands: Roles in structural stabilization and macromolecular interactions. J. Inorg. Biochem..

[10] Eriksson S.E., Ceder S., Bykov V.J.N., Wiman K.G. (2019). p53 as a hub in cellular redox regulation and therapeutic target in cancer. J. Mol. Cell Biol..

[11] Buberg M.L., Witsø I.L., L’Abée-Lund T.M., Wasteson Y. (2020). Zinc and Copper Reduce Conjugative Transfer of Resistance Plasmids from Extended-Spectrum Beta-Lactamase-Producing Escherichia coli. Microb. Drug Resist..

[12] Blencowe D.K., Morby A.P. (2003). Zn(II) metabolism in prokaryotes. FEMS Microbiol. Rev..

[13] Eide D. (1997). Molecular biology of iron and zinc uptake in eukaryotes. Curr. Opin. Cell Biol..

[14] Hennigar S.R., McClung J.P. (2016). Nutritional immunity: Starving pathogens of trace minerals. Am. J. Lifestyle Med..

[15] Wooldridge K.G., Williams P.H. (1993). Iron uptake mechanisms of pathogenic bacteria. FEMS Microbiol. Rev..

[16] Weinberg E.D. (1975). Nutritional immunity. Host’s attempt to withold iron from microbial invaders. JAMA.

[17] Schade A.L., Caroline L. (1944). Raw hen egg white and the role of iron in growth inhibition of Shigella dysenteriae, Staphylococcus aureus, Escherichia coli and Saccharomyces cerevisiae. Science.

[18] Schade A.L., Caroline L. (1946). An Iron-binding Component in Human Blood Plasma. Science.

[19] Weinberg E.D. (1984). Iron withholding: A defense against infection and neoplasia. Physiol. Rev..

[20] Weinberg E.D. (1992). Iron depletion: A defense against intracellular infection and neoplasia. Life Sci..

[21] Kehl-Fie T.E., Skaar E.P. (2010). Nutritional immunity beyond iron: A role for manganese and zinc. Curr. Opin. Chem. Biol..

[22] Corbin B.D., Seeley E.H., Raab A., Feldmann J., Miller M.R., Torres V.J., Anderson K.L., Dattilo B.M., Dunman P.M., Gerads R. (2008). Metal chelation and inhibition of bacterial growth in tissue abscesses. Science.

[23] Hood M.I., Mortensen B.L., Moore J.L., Zhang Y., Kehl-Fie T.E., Sugitani N., Chazin W.J., Caprioli R.M., Skaar E.P. (2012). Identification of an Acinetobacter baumannii zinc acquisition system that facilitates resistance to calprotectin-mediated zinc sequestration. PLoS Pathog..

[24] Hood M.I., Skaar E.P. (2012). Nutritional immunity: Transition metals at the pathogen–host interface. Nat. Rev. Genet..

[25] Lonergan Z.R., Skaar E.P. (2019). Nutrient Zinc at the Host–Pathogen Interface. Trends Biochem. Sci..

[26] Craig G.M., Evans S.J., Brayshaw B.J., Raina S.K. (1990). A study of serum zinc, albumin, alpha-2-macroglobulin and transferrin levels in acute and long stay elderly hospital patients. Postgrad. Med. J..

[27] Foote J.W., Delves H.T. (1984). Albumin bound and alpha 2-macroglobulin bound zinc concentrations in the sera of healthy adults. J. Clin. Pathol..

[28] Foote J.W., Delves H.T. (1984). Distribution of zinc amongst human serum globulins determined by gel filtration-affinity chromatography and atomic-absorption spectrophotometry. Analyst.

[29] Zygiel E.M., Nolan E.M. (2018). Transition Metal Sequestration by the Host-Defense Protein Calprotectin. Annu. Rev. Biochem..

[30] Damo S.M., Kehl-Fie T.E., Sugitani N., Holt M.E., Rathi S., Murphy W.J., Zhang Y., Betz C., Hench L., Günter F. (2013). Molecular basis for manganese sequestration by calprotectin and roles in the innate immune response to invading bacterial pathogens. Proc. Natl. Acad. Sci. USA.

[31] Nakashige T.G., Stephan J.R., Cunden L.S., Brophy M.B., Wommack A.J., Keegan B.C., Shearer J.M., Nolan E.M. (2016). The hexahistidine motif of host-defense protein human calprotectin contributes to zinc withholding and its functional versatility. J. Am. Chem. Soc..

[32] Stephan J.R., Nolan E.M. (2016). Calcium-induced tetramerization and zinc chelation shield human calprotectin from degradation by host and bacterial extracellular proteases. Chem. Sci..

[33] Sohnle P.G., Collins-Lech C., Wiessner J.H. (1991). The Zinc-Reversible Antimicrobial Activity of Neutrophil Lysates and Abscess Fluid Supernatants. J. Infect. Dis..

[34] Sohnle P.G., Collins-Lech C., Wiessner J.H. (1991). Antimicrobial Activity of an Abundant Calcium-Binding Protein in the Cytoplasm of Human Neutrophils. J. Infect. Dis..

[35] Steinbakk M., Naess-Andresen C.F., Lingaas E., Dale I., Brandtzaeg P., Fagerhol M.K. (1990). Antimicrobial actions of calcium binding leucocyte L1 protein, calprotectin. Lancet.

[36] Haase H., Rink L. (2014). Multiple impacts of zinc on immune function. Metallomics.

[37] Haase H., Rink L. (2014). Zinc signals and immune function. BioFactors.

[38] Achouiti A., Vogl T., Urban C.F., Rohm M., Hommes T.J., van Zoelen M.A., Florquin S., Roth J., van ’t Veer C., de Vos A.F. (2012). Myeloid-related protein-14 contributes to protective immunity in gram-negative pneumonia derived sepsis. PLoS Pathog..

[39] Lopez C.A., Beavers W.N., Weiss A., Knippel R.J., Zackular J.P., Chazin W., Skaar E.P. (2019). The immune protein calprotectin impacts Clostridioides difficile metabolism through zinc limitation. MBio.

[40] Cunden L.S., Brophy M.B., Rodriguez G.E., Flaxman H.A., Nolan E.M. (2017). Biochemical and functional evaluation of the intramolecular disulfide bonds in the zinc-chelating antimicrobial protein human S100A7 (psoriasin). Biochemistry.

[41] Crane J.K., Broome J.E., Reddinger R.M., Werth B.B. (2014). Zinc protects against Shiga-toxigenic Escherichia coli by acting on host tissues as well as on bacteria. BMC Microbiol..

[42] Medeiros P., Bolick D.T., Roche J.K., Noronha F., Pinheiro C., Kolling G.L., Lima A., Guerrant R.L. (2013). The micronutrient zinc inhibits EAEC strain 042 adherence, biofilm formation, virulence gene expression, and epithelial cytokine responses benefiting the infected host. Virulence.

[43] Hunter J., Arentz S., Goldenberg J., Yang G., Beardsley J., Mertz D., Leeder S. (2020). Rapid review protocol: Zinc for the prevention or treatment of COVID-19 and other coronavirus-related respiratory tract infections. Integr. Med. Res..

[44] te Velthuis A.J., van den Worm S.H., Sims A.C., Baric R.S., Snijder E.J., van Hemert M.J. (2010). Zn(2+) inhibits coronavirus and arterivirus RNA polymerase activity in vitro and zinc ionophores block the replication of these viruses in cell culture. PLoS Pathog..

[45] Cordingley M.G., Register R.B., Callahan P.L., Garsky V.M., Colonno R.J. (1989). Cleavage of small peptides in vitro by human rhinovirus 14 3C protease expressed in Escherichia coli. J. Virol..

[46] Fenstermacher K.J., DeStefano J.J. (2011). Mechanism of HIV reverse transcriptase inhibition by zinc: Formation of a highly stable enzyme-(primer-template) complex with profoundly diminished catalytic activity. J. Biol. Chem..

[47] Crane J.K., Cheema M.B., Olyer M.A., Sutton M.D. (2018). Zinc blockade of SOS response inhibits horizontal transfer of antibiotic resistance genes in enteric bacteria. Front. Cell Infect. Microbiol..

[48] Bunnell B.E., Escobar J.F., Bair K.L., Sutton M.D., Crane J.K. (2017). Zinc blocks SOS-induced antibiotic resistance via inhibition of RecA in Escherichia coli. PLoS ONE.

[49] Botella H., Peyron P., Levillain F., Poincloux R., Poquet Y., Brandli I., Wang C., Tailleux L., Tilleul S., Charriere G.M. (2011). Mycobacterial p(1)-type ATPases mediate resistance to zinc poisoning in human macrophages. Cell Host Microbe..

[50] Ong C.L., Gillen C.M., Barnett T.C., Walker M.J., McEwan A.G. (2014). An antimicrobial role for zinc in innate immune defense against group A streptococcus. J. Infect. Dis..

[51] Kapetanovic R., Bokil N.J., Achard M.E., Ong C.L., Peters K.M., Stocks C.J., Phan M.D., Monteleone M., Schroder K., Irvine K.M. (2016). Salmonella employs multiple mechanisms to subvert the TLR-inducible zinc-mediated antimicrobial response of human macrophages. FASEB J..

[52] McDevitt C.A., Ogunniyi A.D., Valkov E., Lawrence M.C., Kobe B., McEwan A.G., Paton J.C. (2011). A molecular mechanism for bacterial susceptibility to zinc. PLoS Pathog..

[53] Botella H., Stadthagen G., Lugo-Villarino G., De Chastellier C., Neyrolles O. (2012). Metallobiology of host–pathogen interactions: An intoxicating new insight. Trends Microbiol..

[54] Eijkelkamp B.A., Morey J.R., Ween M.P., Ong C.L., McEwan A.G., Paton J.C., McDevitt C.A. (2014). Extracellular zinc competitively inhibits manganese uptake and compromises oxidative stress management in Streptococcus pneumoniae. PLoS ONE.

[55] Ong C.-L.Y., Walker M.J., McEwan A.G. (2015). Zinc disrupts central carbon metabolism and capsule biosynthesis in Streptococcus pyogenes. Sci. Rep..

[56] Makthal N., Kumaraswami M. (2017). Zinc’ing it out: Zinc homeostasis mechanisms and their impact on the pathogenesis of human pathogen group A streptococcus. Metallomics.

[57] Crane J.K., Olson R.A., Jones H.M., Duffey M.E. (2002). Release of ATP during host cell killing by enteropathogenic E. coli and its role as a secretory mediator. Am. J. Physiol. Gastrointest. Liver Physiol..

[58] Crane J.K., Shulgina I., Naeher T.M. (2007). Ecto-5′-nucleotidase and intestinal ion secretion by enteropathogenic Escherichia coli. Purinergic Signal..

[59] Barffour M.A., Hinnouho G.M., Wessells K.R., Kounnavong S., Ratsavong K., Sitthideth D., Bounheuang B., Sengnam K., Chanhthavong B., Arnold C.D. (2020). Effects of therapeutic zinc supplementation for diarrhea and two preventive zinc supplementation regimens on the incidence and duration of diarrhea and acute respiratory tract infections in rural Laotian children: A randomized controlled trial. J. Glob. Health.

[60] Bhutta Z.A., Bird S.M., Black R.E., Brown K.H., Gardner J.M., Hidayat A., Khatun F., Martorell R., Ninh N.X., Penny M.E. (2000). Therapeutic effects of oral zinc in acute and persistent diarrhea in children in developing countries: Pooled analysis of randomized controlled trials. Am. J. Clin. Nutr..

[61] Metzler-Zebeli B.U., Caine W.R., McFall M., Miller B., Ward T.L., Kirkwood R.N., Mosenthin R. (2010). Supplementation of diets for lactating sows with zinc amino acid complex and gastric nutriment-intubation of suckling pigs with zinc methionine on mineral status, intestinal morphology and bacterial translocation in lipopolysaccharide-challenged weaned pigs. J. Anim. Physiol. Anim. Nutr. (Berl).

[62] Crane J.K., Naeher T.M., Shulgina I., Zhu C., Boedeker E.C. (2007). Effect of zinc in enteropathogenic Escherichia coli infection. Infect. Immun..

[63] Magallon J., Chiem K., Tran T., Ramirez M.S., Jimenez V., Tolmasky M.E. (2019). Restoration of susceptibility to amikacin by 8-hydroxyquinoline analogs complexed to zinc. PLoS ONE.

[64] Crane J.K., Byrd I.W., Boedeker E.C. (2011). *Virulence* Inhibition by Zinc in Shiga-ToxigenicEscherichia coli. Infect. Immun..

[65] Cirz R.T., Romesberg F.E. (2006). Induction and Inhibition of Ciprofloxacin Resistance-Conferring Mutations in Hypermutator Bacteria. Antimicrob. Agents Chemother..

[66] Recacha E., Machuca J., De Alba P.D., Ramos-Güelfo M., Pérez F.M.D., Beltrán J.R., Blázquez J., Pascual A., Martínez J.M.R. (2017). Quinolone Resistance Reversion by Targeting the SOS Response. MBio.

[67] Plaut R.D., Beaber J.W., Zemansky J., Kaur A.P., George M., Biswas B., Henry M., Bishop-Lilly K.A., Mokashi V., Hannah R.M. (2014). Genetic evidence for the involvement of the S-layer protein gene sap and the sporulation genes spo0A, spo0B, and spo0F in phage AP50c infection of Bacillus anthracis. J. Bacteriol..

[68] Lin D.L., Tran T., Alam J.Y., Herron S.R., Ramirez M.S., Tolmasky M.E. (2014). Inhibition of aminoglycoside 6’-N-acetyltransferase type Ib by zinc: Reversal of amikacin resistance in Acinetobacter baumannii and Escherichia coli by a zinc ionophore. Antimicrob. Agents Chemother..

[69] Kelly P., Besa E., Zyambo K., Louis-Auguste J., Lees J., Banda T., Soko R., Banda R., Amadi B., Watson A. (2016). Endomicroscopic and transcriptomic analysis of impaired barrier function and Mmalabsorption in environmental enteropathy. PLoS Negl. Trop. Dis..

[70] Korpe P.S., Petri W.A. (2012). Environmental enteropathy: Critical implications of a poorly understood condition. Trends Mol. Med..

[71] Choudhry N., Scott F., Edgar M., Sanger G.J., Kelly P. (2021). Reversal of pathogen-induced barrier defects in intestinal epithelial cells by contra-pathogenicity agents. Dig. Dis. Sci..

[72] Wu C., Labrie J., Tremblay Y.D., Haine D., Mourez M., Jacques M. (2013). Zinc as an agent for the prevention of biofilm formation by pathogenic bacteria. J. Appl. Microbiol..

[73] Kolodziejczak-Radzimska A., Jesionowski T. (2014). Zinc oxide-from synthesis to application: A review. Materials.

[74] Morshedtalab Z., Rahimi G., Emami-Nejad A., Farasat A., Mohammadbeygi A., Ghaedamini N., Negahdary M. (2020). Antibacterial assessment of zinc sulfide nanoparticles against Streptococcus pyogenes and Acinetobacter baumannii. Curr. Top Med. Chem..

[75] Sanchez-Lopez E., Gomes D., Esteruelas G., Bonilla L., Lopez-Machado A.L., Galindo R., Cano A., Espina M., Ettcheto M., Camins A. (2020). Metal-based nanoparticles as antimicrobial agents: An overview. NanoMaterials.

[76] Ye Q., Chen W., Huang H., Tang Y., Wang W., Meng F., Wang H., Zheng Y. (2020). Iron and zinc ions, potent weapons against multidrug-resistant bacteria. Appl. Microbiol. Biotechnol..

[77] Slavin Y.N., Asnis J., Hafeli U.O., Bach H. (2017). Metal nanoparticles: Understanding the mechanisms behind antibacterial activity. J. Nanobiotechnol..

[78] Stensberg M.C., Wei Q., McLamore E.S., Porterfield D.M., Wei A., Sepulveda M.S. (2011). Toxicological studies on silver nanoparticles: Challenges and opportunities in assessment, monitoring and imaging. Nanomedicine (Lond).

[79] Vijayakumar S., Krishnakumar C., Arulmozhi P., Mahadevan S., Parameswari N. (2018). Biosynthesis, characterization and antimicrobial activities of zinc oxide nanoparticles from leaf extract of Glycosmis pentaphylla (Retz.) DC. Microb. Pathog..

[80] Singh P., Nanda A. (2013). Antimicrobial and antifungal potential of zinc oxide nanoparticles in comparison to conventional zinc oxide particles. J. Chem. Pharm. Res..

[81] Yu J., Zhang W., Li Y., Wang G., Yang L., Jin J., Chen Q., Huang M. (2014). Synthesis, characterization, antimicrobial activity and mechanism of a novel hydroxyapatite whisker/nano zinc oxide biomaterial. Biomed. Mater..

[82] Sirelkhatim A., Mahmud S., Seeni A., Kaus N.H.M., Ann L.C., Bakhori S.K.M., Hasan H., Mohamad D. (2015). Review on zinc oxide nanoparticles: Antibacterial activity and toxicity mechanism. Nanomicro. Lett..

[83] Sukri S., Shameli K., Wong M., Teow S., Ismail N. (2019). Cytotoxicity and antibacterial activities of plant-mediated synthesized zinc oxide (ZnO) nanoparticles using Punica granatum (pomegranate) fruit peels extract. J. Mol. Struct..

[84] Happy A., Soumya M., Venkat Kumar S., Rajeshkumar S., Sheba R.D., Lakshmi T., Deepak Nallaswamy V. (2019). Phyto-assisted synthesis of zinc oxide nanoparticles using Cassia alata and its antibacterial activity against Escherichia coli. Biochem. Biophys. Rep..

[85] Hantke K. (2005). Bacterial zinc uptake and regulators. Curr. Opin. Microbiol..

[86] Waldron K.J., Robinson N.J. (2009). How do bacterial cells ensure that metalloproteins get the correct metal?. Nat. Rev. Genet..

[87] Chandrangsu P., Rensing C., Helmann J.D. (2017). Metal homeostasis and resistance in bacteria. Nat. Rev. Genet..

[88] Outten C.E., O’Halloran T.V. (2001). Femtomolar sensitivity of metalloregulatory proteins controlling zinc homeostasis. Science.

[89] Waldron K.J., Rutherford J.C., Ford D., Robinson N.J. (2009). Metalloproteins and metal sensing. Nature.

[90] Grim K.P., Radin J.N., Solorzano P.K.P., Morey J.R., Frye K.A., Ganio K., Neville S.L., McDevitt C.A., Kehl-Fie T.E. (2020). Intracellular accumulation of staphylopine can sensitize Staphylococcus aureus to host-imposed zinc starvation by chelation-independent toxicity. J. Bacteriol..

[91] Capdevila D.A., Wang J., Giedroc D.P. (2016). Bacterial Strategies to Maintain Zinc Metallostasis at the Host-Pathogen Interface. J. Biol. Chem..

[92] Blindauer C.A. (2015). Advances in the molecular understanding of biological zinc transport. Chem. Commun..

[93] Hesse L.E., Lonergan Z.R., Beavers W.N., Skaar E.P. (2019). The Acinetobacter baumannii Znu system overcomes host-imposed nutrient zinc limitation. Infect. Immun..

[94] Lonergan Z.R., Nairn B.L., Wang J., Hsu Y.P., Hesse L.E., Beavers W.N., Chazin W.J., Trinidad J.C., VanNieuwenhze M.S., Giedroc D.P. (2019). An Acinetobacter baumannii, zinc-regulated peptidase maintains cell wall integrity during immune-mediated nutrient sequestration. Cell Rep..

[95] Schneider E., Hunke S. (1998). ATP-binding-cassette (ABC) transport systems: Functional and structural aspects of the ATP-hydrolyzing subunits/domains. FEMS Microbiol. Rev..

[96] Patzer S.I., Hantke K. (1998). The ZnuABC high-affinity zinc uptake system and its regulator Zur in Escherichia coli. Mol. Microbiol..

[97] Berntsson R.P., Smits S.H., Schmitt L., Slotboom D.J., Poolman B. (2010). A structural classification of substrate-binding proteins. FEBS Lett..

[98] van der Heide T., Poolman B. (2002). ABC transporters: One, two or four extracytoplasmic substrate-binding sites?. EMBO Rep..

[99] Warner D.M., Levy S.B. (2011). SoxS Increases the Expression of the Zinc Uptake System ZnuACB in an Escherichia coli Murine Pyelonephritis Model. J. Bacteriol..

[100] Panina E.M., Mironov A.A., Gelfand M.S. (2003). Comparative genomics of bacterial zinc regulons: Enhanced ion transport, pathogenesis, and rearrangement of ribosomal proteins. Proc. Natl. Acad. Sci. USA.

[101] Bellotti D., Rowinska-Zyrek M., Remelli M. (2020). Novel insights into the metal binding ability of ZinT periplasmic protein from Escherichia coli and Salmonella enterica. Dalton Trans..

[102] Gabbianelli R., Scotti R., Ammendola S., Petrarca P., Nicolini L., Battistoni A. (2011). Role of ZnuABC and ZinT in Escherichia coli O157:H7 zinc acquisition and interaction with epithelial cells. BMC Microbiol..

[103] Hubert K., Devos N., Mordhorst I., Tans C., Baudoux G., Feron C., Goraj K., Tommassen J., Vogel U., Poolman J.T. (2013). ZnuD, a potential candidate for a simple and universal Neisseria meningitidis vaccine. Infect. Immun..

[104] Stork M., Grijpstra J., Bos M.P., Manas Torres C., Devos N., Poolman J.T., Chazin W.J., Tommassen J. (2013). Zinc piracy as a mechanism of Neisseria meningitidis for evasion of nutritional immunity. PLoS Pathog..

[105] Di Lorenzo M., Stork M., Tolmasky M.E., Alonso J. (2014). Plasmid-Encoded Iron Uptake Systems. Plasmids. Biology and Impact in Biotechnology and Discovery.

[106] Stojnev T., Harichova J., Ferianc P., Nystrom T. (2007). Function of a novel cadmium-induced yodA protein in Escherichia coli. Curr. Microbiol..

[107] Calmettes C., Ing C., Buckwalter C.M., El Bakkouri M., Chieh-Lin Lai C., Pogoutse A., Gray-Owen S.D., Pomes R., Moraes T.F. (2015). The molecular mechanism of Zinc acquisition by the neisserial outer-membrane transporter ZnuD. Nat. Commun..

[108] Pederick V.G., Eijkelkamp B.A., Begg S.L., Ween M.P., McAllister L.J., Paton J.C., McDevitt C.A. (2015). ZnuA and zinc homeostasis in Pseudomonas aeruginosa. Sci. Rep..

[109] Qamsari M.M., Rasooli I., Chaudhuri S., Astaneh S.D.A., Schryvers A.B. (2020). Hybrid antigens expressing surface loops of ZnuD from Acinetobacter baumannii Is capable of inducing protection against infection. Front. Immunol..

[110] Plumptre C.D., Eijkelkamp B.A., Morey J.R., Behr F., Counago R.M., Ogunniyi A.D., Kobe B., O’Mara M.L., Paton J.C., McDevitt C.A. (2014). AdcA and AdcAII employ distinct zinc acquisition mechanisms and contribute additively to zinc homeostasis in Streptococcus pneumoniae. Mol. Microbiol..

[111] Chu B.C., Garcia-Herrero A., Johanson T.H., Krewulak K.D., Lau C.K., Peacock R.S., Slavinskaya Z., Vogel H.J. (2010). Siderophore uptake in bacteria and the battle for iron with the host; a bird’s eye view. Biometals.

[112] Ghssein G., Brutesco C., Ouerdane L., Fojcik C., Izaute A., Wang S., Hajjar C., Lobinski R., Lemaire D., Richaud P. (2016). Biosynthesis of a broad-spectrum nicotianamine-like metallophore in Staphylococcus aureus. Science.

[113] Grim K.P., San Francisco B., Radin J.N., Brazel E.B., Kelliher J.L., Parraga Solorzano P.K., Kim P.C., McDevitt C.A., Kehl-Fie T.E. (2017). The metallophore staphylopine enables Staphylococcus aureus to compete with the host for zinc and overcome nutritional immunity. MBio.

[114] Lhospice S., Gomez N.O., Ouerdane L., Brutesco C., Ghssein G., Hajjar C., Liratni A., Wang S., Richaud P., Bleves S. (2017). Pseudomonas aeruginosa zinc uptake in chelating environment is primarily mediated by the metallophore pseudopaline. Sci. Rep..

[115] McFarlane J.S., Lamb A.L. (2017). Biosynthesis of an Opine Metallophore byPseudomonas aeruginosa. Biochemistry.

[116] Mastropasqua M.C., D’Orazio M., Cerasi M., Pacello F., Gismondi A., Canini A., Canuti L., Consalvo A., Ciavardelli D., Chirullo B. (2017). Growth of Pseudomonas aeruginosa in zinc poor environments is promoted by a nicotianamine-related metallophore. Mol. Microbiol..

[117] Lin W., Chai J., Love J., Fu D. (2010). Selective electrodiffusion of zinc ions in a Zrt-, Irt-like protein, ZIPB. J. Biol. Chem..

[118] Hudek L., Pearson L.A., Michalczyk A., Neilan B.A., Ackland M.L. (2013). Functional characterization of the twin ZIP/SLC39 metal transporters, NpunF3111 and NpunF2202 in Nostoc punctiforme. Appl. Microbiol. Biotechnol..

[119] Cerasi M., Liu J.Z., Ammendola S., Poe P., Petrarca A.J., Pesciaroli M., Pasquali P., Raffatellu M., Battistoni A. (2014). The ZupT transporter plays an important role in zinc homeostasis and contributes to Salmonella enterica *Virulence*. Metallomics.

[120] Grass G., Franke S., Taudte N., Nies D.H., Kucharski L.M., Maguire M.E., Rensing C. (2005). The metal permease ZupT from Escherichia coli is a transporter with a broad substrate spectrum. J. Bacteriol..

[121] Kolaj-Robin O., Russell D., Hayes K.A., Pembroke J.T., Soulimane T. (2015). Cation diffusion facilitator family: Structure and function. FEBS Lett..

[122] Nies D.H. (2003). Efflux-mediated heavy metal resistance in prokaryotes. FEMS Microbiol. Rev..

[123] Nairn B.L., Lonergan Z.R., Wang J., Braymer J.J., Zhang Y., Calcutt M.W., Lisher J.P., Gilston B.A., Chazin W.J., de Crecy-Lagard V. (2016). The response of Acinetobacter baumannii to zinc starvation. Cell Host Microbe..

[124] Montanini B., Blaudez D., Jeandroz S., Sanders D., Chalot M. (2007). Phylogenetic and functional analysis of the Cation Diffusion Facilitator (CDF) family: Improved sig*Nature* and prediction of substrate specificity. BMC Genomics.

[125] Udagedara S.R., La Porta D.M., Spehar C., Purohit G., Hein M.J.A., Fatmous M.E., Casas Garcia G.P., Ganio K., McDevitt C.A., Maher M.J. (2020). Structural and functional characterizations of the C-terminal domains of CzcD proteins. J. Inorg. Biochem..

[126] Bublitz M., Morth J.P., Nissen P. (2011). P-type ATPases at a glance. J. Cell Sci..

[127] Klein J.S., Lewinson O. (2011). Bacterial ATP-driven transporters of transition metals: Physiological roles, mechanisms of action, and roles in bacterial *Virulence*. Metallomics.

[128] Wang K., Sitsel O., Meloni G., Autzen H.E., Andersson M., Klymchuk T., Nielsen A.M., Rees D.C., Nissen P., Gourdon P. (2014). Structure and mechanism of Zn^2+^-transporting P-type ATPases. Nature.

[129] Raimunda D., Subramanian P., Stemmler T., Arguello J.M. (2012). A tetrahedral coordination of Zinc during transmembrane transport by P-type Zn(2+)-ATPases. Biochim. Biophys. Acta.

[130] Okkeri J., Haltia T. (2006). The metal-binding sites of the zinc-transporting P-type ATPase of Escherichia coli. Lys693 and Asp714 in the seventh and eighth transmembrane segments of ZntA contribute to the coupling of metal binding and ATPase activity. Biochim. Et Biophys. Acta (Bba) Gen. Subj..

[131] Argüello J.M., González-Guerrero M., Raimunda D. (2011). Bacterial Transition Metal P1B-ATPases: Transport Mechanism and Roles in *Virulence*. Biochemistry.

[132] Smith A.T., Smith K.P., Rosenzweig A.C. (2014). Diversity of the metal-transporting P1B-type ATPases. JBIC J. Biol. Inorg. Chem..

[133] Kuroda M., Hayashi H., Ohta T. (1999). Chromosome-determined zinc-responsible operon czr in Staphylococcus aureus strain. Microbiol. Immunol..

[134] Grass G., Fan B., Rosen B.P., Franke S., Nies D.H., Rensing C. (2001). ZitB (YbgR), a member of the cation diffusion facilitator family, is an additional zinc transporter in Escherichia coli. J. Bacteriol..

[135] Chao Y., Fu D. (2004). Kinetic Study of the Antiport Mechanism of an Escherichia coli Zinc Transporter, ZitB. J. Biol. Chem..

[136] Anton A., Weltrowski A., Haney C.J., Franke S., Grass G., Rensing C., Nies D.H. (2004). Characteristics of zinc transport by two bacterial cation diffusion facilitators from Ralstonia metallidurans CH34 and Escherichia coli. J. Bacteriol..

[137] Coudray N., Valvo S., Hu M., Lasala R., Kim C., Vink M., Zhou M., Provasi D., Filizola M., Tao J. (2013). Inward-facing conformation of the zinc transporter YiiP revealed by cryoelectron microscopy. Proc. Natl. Acad. Sci. USA.

[138] Lee S.M., Grass G., Haney C.J., Fan B., Rosen B.P., Anton A., Nies D.H., Rensing C. (2002). Functional analysis of the Escherichia coli zinc transporter ZitB. FEMS Microbiol. Lett..

[139] Guffanti A.A., Wei Y., Rood S.V., Krulwich T.A. (2002). An antiport mechanism for a member of the cation diffusion facilitator family: Divalent cations efflux in exchange for K+ and H+. Mol. Microbiol..

[140] Lu M., Chai J., Fu D. (2009). Structural basis for autoregulation of the zinc transporter YiiP. Nat. Struct. Mol. Biol..

[141] Lu M., Fu D. (2007). Structure of the Zinc Transporter YiiP. Science.

[142] Cherezov V., Hofer N., Szebenyi D.M., Kolaj O., Wall J.G., Gillilan R., Srinivasan V., Jaroniec C.P., Caffrey M. (2008). Insights into the mode of action of a putative zinc transporter CzrB in Thermus thermophilus. Structure.

[143] Higuchi T., Hattori M., Tanaka Y., Ishitani R., Nureki O. (2009). Crystal structure of the cytosolic domain of the cation diffusion facilitator family protein. Proteins.

[144] Zeytuni N., Uebe R., Maes M., Davidov G., Baram M., Raschdorf O., Nadav-Tsubery M., Kolusheva S., Bitton R., Goobes G. (2014). Cation diffusion facilitators transport initiation and regulation is mediated by cation induced conformational changes of the cytoplasmic domain. PLoS ONE.

[145] Russell D., Soulimane T. (2012). Evidence for zinc and cadmium binding in a CDF transporter lacking the cytoplasmic domain. FEBS Lett..

[146] Prince R.W., Cox C.D., Vasil M.L. (1993). Coordinate regulation of siderophore and exotoxin A production: Molecular cloning and sequencing of the Pseudomonas aeruginosa fur gene. J. Bacteriol..

[147] Kim E.-H., Nies D.H., McEvoy M.M., Rensing C. (2011). Switch or Funnel: How RND-Type Transport Systems Control Periplasmic Metal Homeostasis. J. Bacteriol..

[148] Alquethamy S.F., Adams F.G., Naidu V., Khorvash M., Pederick V.G., Zang M., Paton J.C., Paulsen I.T., Hassan K.A., Cain A.K. (2020). The Role of zinc efflux during Acinetobacter baumannii infection. ACS Infect Dis..

[149] Meni A., Yukl E.T. (2020). Structural Features Mediating Zinc Binding and Transfer in the AztABCD Zinc Transporter System. Biomolecules.

[150] Dyla M., Hansen S.B., Nissen P., Kjaergaard M. (2019). Structural dynamics of P-type ATPase ion pumps. Biochem. Soc. Trans..

[151] Bagg A., Neilands J.B. (1987). Ferric uptake regulation protein acts as a repressor, employing iron(II) as a cofactor to bind the operator of an iron transport operon in Escherichia coli. Biochemistry.

[152] Hantke K. (1987). Selection procedure for deregulated iron transport mutants (fur) in Escherichia coli K 12: Fur not only affects iron metabolism. Mol. Genet. Genom..

[153] Pinochet-Barros A., Helmann J.D. (2018). Redox Sensing by Fe2+in Bacterial Fur Family Metalloregulators. Antioxid. Redox Signal..

[154] Tolmasky M.E., Wertheimer A.M., Actis L.A., Crosa J.H. (1994). Characterization of the Vibrio anguillarum fur gene: Role in regulation of expression of the FatA outer membrane protein and catechols. J. Bacteriol..

[155] Staggs T.M., Perry R.D. (1991). Identification and cloning of a fur regulatory gene in Yersinia pestis. J. Bacteriol..

[156] Litwin C.M., Boyko S.A., Calderwood S.B. (1992). Cloning, sequencing, and transcriptional regulation of the Vibrio cholerae fur gene. J. Bacteriol..

[157] Daniel C., Haentjens S., Bissinger M.C., Courcol R.J. (1999). Characterization of the Acinetobacter baumannii Fur regulator: Cloning and sequencing of the fur homolog gene. FEMS Microbiol. Lett..

[158] Hantke K. (2001). Iron and metal regulation in bacteria. Curr. Opin. Microbiol..

[159] Coy M., Neilands J.B. (1991). Structural dynamics and functional domains of the Fur protein. Biochemistry.

[160] Mills S.A., Marletta M.A. (2005). Metal Binding Characteristics and Role of Iron Oxidation in the Ferric Uptake Regulator fromEscherichia coli. Biochemistry.

[161] Waldbeser L.S., Tolmasky M.E., Actis L.A., Crosa J.H. (1993). Mechanisms for negative regulation by iron of the fatA outer membrane protein gene expression in Vibrio anguillarum. J. Biol. Chem..

[162] Salinas P.C., Tolmasky M.E., Crosa J.H. (1989). Regulation of the iron uptake system in Vibrio anguillarum: Evidence for a cooperative effect between two transcriptional activators. Proc. Natl. Acad. Sci. USA.

[163] Troxell B., Hassan H.M. (2013). Transcriptional regulation by Ferric Uptake Regulator (Fur) in pathogenic bacteria. Front. Cell Infect. Microbiol..

[164] Fillat M.F. (2014). The FUR (ferric uptake regulator) superfamily: Diversity and versatility of key transcriptional regulators. Arch. Biochem. Biophys..

[165] Huang D.L., Tang D.J., Liao Q., Li H.C., Chen Q., He Y.Q., Feng J.X., Jiang B.L., Lu G.T., Chen B. (2008). The Zur of Xanthomonas campestris functions as a repressor and an activator of putative zinc homeostasis genes via recognizing two distinct sequences within its target promoters. Nucleic Acids Res..

[166] Gilston B.A., Wang S., Marcus M.D., Canalizo-Hernandez M.A., Swindell E.P., Xue Y., Mondragon A., O’Halloran T.V. (2014). Structural and mechanistic basis of zinc regulation across the E. coli Zur regulon. PLoS Biol..

[167] Lucarelli D., Russo S., Garman E., Milano A., Meyer-Klaucke W., Pohl E. (2007). Crystal structure and function of the zinc uptake regulator FurB from Mycobacterium tuberculosis. J. Biol. Chem..

[168] Shin J.H., Jung H.J., An Y.J., Cho Y.B., Cha S.S., Roe J.H. (2011). Graded expression of zinc-responsive genes through two regulatory zinc-binding sites in Zur. Proc. Natl. Acad. Sci. USA.

[169] Mortensen B.L., Rathi S., Chazin W.J., Skaar E.P. (2014). Acinetobacter baumannii response to host-mediated zinc limitation requires the transcriptional regulator Zur. J. Bacteriol..

[170] Lindsay J.A., Foster S.J. (2001). Zur: A Zn(2+)-responsive regulatory element of Staphylococcus aureus. Microbiology (Reading).

[171] Sanson M., Makthal N., Flores A.R., Olsen R.J., Musser J.M., Kumaraswami M. (2015). Adhesin competence repressor (AdcR) from Streptococcus pyogenes controls adaptive responses to zinc limitation and contributes to *Virulence*. Nucleic Acids Res..

[172] Makthal N., Do H., Wendel B.M., Olsen R.J., Helmann J.D., Musser J.M., Kumaraswami M. (2020). Group A Streptococcus AdcR regulon participates in bacterial defense against host-mediated zinc sequestration and contributes to *Virulence*. Infect. Immun..

[173] Reyes-Caballero H., Guerra A.J., Jacobsen F.E., Kazmierczak K.M., Cowart D., Koppolu U.M., Scott R.A., Winkler M.E., Giedroc D.P. (2010). The metalloregulatory zinc site in Streptococcus pneumoniae AdcR, a zinc-activated MarR family repressor. J. Mol. Biol..

[174] Toewiwat N., Whangsuk P., Ploypradith W., Mongkolsuk S., Loprasert S. (2020). Cefoperazone induces esterase B expression by EstR and esterase B enhances cefoperazone activity at the periplasm. Int. J. Med. Microbiol..

[175] Deng X., Li M., Liu L., Zhang J., Zhang Y., Guo J., Zhao T., Cao S., Li Z., Zhang H. (2020). Functional analysis of Brucella reveals transcriptional regulation of MarR. Microb. Pathog..

[176] Guerra A.J., Dann C.E., Giedroc D.P. (2011). Crystal structure of the zinc-dependent MarR family transcriptional regulator AdcR in the Zn(II)-bound state. J. Am. Chem. Soc..

[177] Martin J.E., Edmonds K.A., Bruce K.E., Campanello G.C., Eijkelkamp B.A., Brazel E.B., McDevitt C.A., Winkler M.E., Giedroc D.P. (2017). The zinc efflux activator SczA protects Streptococcus pneumoniae serotype 2 D39 from intracellular zinc toxicity. Mol. Microbiol..

[178] Kloosterman T.G., van der Kooi-Pol M.M., Bijlsma J.J., Kuipers O.P. (2007). The novel transcriptional regulator SczA mediates protection against Zn^2+^ stress by activation of the Zn^2+^-resistance gene czcD in Streptococcus pneumoniae. Mol. Microbiol..

[179] Canneva F., Branzoni M., Riccardi G., Provvedi R., Milano A. (2005). Rv2358 and FurB: Two transcriptional regulators from Mycobacterium tuberculosis which respond to zinc. J. Bacteriol..

[180] Eckelt E., Jarek M., Fromke C., Meens J., Goethe R. (2014). Identification of a lineage specific zinc responsive genomic island in Mycobacterium avium ssp. paratuberculosis. BMC Genom..

[181] Milano A., Branzoni M., Canneva F., Profumo A., Riccardi G. (2004). The Mycobacterium tuberculosis Rv2358-furB operon is induced by zinc. Res. Microbiol..

[182] Ramirez M.S., Nikolaidis N., Tolmasky M.E. (2013). Rise and dissemination of aminoglycoside resistance: The aac(6′)-Ib paradigm. Front. Microbiol..

[183] Gao F., Yan X., Shakya T., Baettig O.M., Ait-Mohand-Brunet S., Berghuis A.M., Wright G.D., Auclair K. (2006). Synthesis and structure-activity relationships of truncated bisubstrate inhibitors of aminoglycoside 6’-N-acetyltransferases. J. Med. Chem..

[184] Vong K., Auclair K. (2012). Understanding and overcoming aminoglycoside resistance caused by N-6′-acetyltransferase. MedChemComm.

[185] Goethe E., Laarmann K., Luhrs J., Jarek M., Meens J., Lewin A., Goethe R. (2020). Critical role of Zur and SmtB in zinc homeostasis of Mycobacterium smegmatis. MSystems.

[186] Houghton J.L., Green K.D., Chen W., Garneau-Tsodikova S. (2010). The future of aminoglycosides: The end or renaissance?. ChemBiochem.

[187] Block M., Blanchard D.L. (2020). Aminoglycosides.

[188] Ramirez M.S., Tolmasky M.E. (2017). Amikacin: Uses, Resistance, and Prospects for Inhibition. Molecules.

[189] Ramirez M.S., Tolmasky M.E. (2010). Aminoglycoside modifying enzymes. Drug Resist. Updat..

[190] Tolmasky M.E., Atta-ur-Rhaman (2017). Strategies to prolong the useful life of existing antibiotics and help overcoming the antibiotic resistance crisis. Frontiers in Clinical Drug Research-Anti Infectives.

[191] Papp-Wallace K.M., Bonomo R.A. (2016). New beta-lactamase inhibitors in the clinic. Infect. Dis. Clin. North Am..

[192] Daigle D.M., McKay G.A., Thompson P.R., Wright G.D. (1999). Aminoglycoside antibiotic phosphotransferases are also serine protein kinases. Chem. Biol..

[193] Chiem K., Fuentes B.A., Lin D.L., Tran T., Jackson A., Ramirez M.S., Tolmasky M.E. (2015). Inhibition of aminoglycoside 6’-N-acetyltransferase type Ib-mediated amikacin resistance in Klebsiella pneumoniae by zinc and copper pyrithione. Antimicrob. Agents Chemother..

[194] Lin D.L., Tran T., Adams C., Alam J.Y., Herron S.R., Tolmasky M.E. (2013). Inhibitors of the aminoglycoside 6’-N-acetyltransferase type Ib [AAC(6’)-Ib] identified by in silico molecular docking. Bioorg. Med. Chem. Lett..

[195] Tran T., Chiem K., Jani S., Arivett B.A., Lin D.L., Lad R., Jimenez V., Farone M.B., Debevec G., Santos R. (2018). Tolmasky. Identification of a small molecule inhibitor of the aminoglycoside 6’-N-acetyltransferase type Ib [AAC(6’)-Ib] using mixture-based combinatorial libraries. Int. J. Antimicrob. Agents.

[196] Li Y., Green K.D., Johnson B.R., Garneau-Tsodikova S. (2015). Inhibition of aminoglycoside acetyltransferase resistance enzymes by metal salts. Antimicrob. Agents Chemother..

[197] Chiem K., Hue F., Magallon J., Tolmasky M.E. (2018). Inhibition of aminoglycoside 6’-N-acetyltransferase type Ib-mediated amikacin resistance by zinc complexed with clioquinol, an ionophore active against tumors and neurodegenerative diseases. Int. J. Antimicrob. Agents.

[198] Ahmed S., Sony S.A., Chowdhury M.B., Ullah M.M., Paul S., Hossain T. (2020). Retention of antibiotic activity against resistant bacteria harbouring aminoglycoside-N-acetyltransferase enzyme by adjuvants: A combination of in-silico and in-vitro study. Sci. Rep..

[199] Singh R., Sripada L., Singh R. (2014). Side effects of antibiotics during bacterial infection: Mitochondria, the main target in host cell. Mitochondrion.

[200] Sensi S.L., Ton-That D., Sullivan P.G., Jonas E.A., Gee K.R., Kaczmarek L.K., Weiss J.H. (2003). Modulation of mitochondrial function by endogenous Zn^2+^ pools. Proc. Natl. Acad. Sci. USA.

[201] Sensi S.L., Yin H.Z., Carriedo S.G., Rao S.S., Weiss J.H. (1999). Preferential Zn^2+^ influx through Ca^2+^-permeable AMPA/kainate channels triggers prolonged mitochondrial superoxide production. Proc. Natl. Acad. Sci. USA.

[202] Frederickson C.J. (1989). Neurobiology of Zinc and Zinc-Containing Neurons. Int. Rev. Neurobiol..

[203] Bettger W.J., O’Dell B.L. (1981). A critical physiological role of zinc in the structure and function of biomembranes. Life Sci..

[204] Vallee B.L., Falchuk K.H. (1993). The biochemical basis of zinc physiology. Physiol. Rev..

[205] Brand I.A., Kleineke J. (1996). Intracellular Zinc Movement and Its Effect on the Carbohydrate Metabolism of Isolated Rat Hepatocytes. J. Biol. Chem..

[206] Simons T.J.B. (1991). Intracellular free zinc and zinc buffering in human red blood cells. J. Membr. Biol..

[207] Simons T.J. (1993). Measurement of free Zn^2+^ ion concentration with the fluorescent probe mag-fura-2 (furaptra). J. Biochem. Biophys. Methods.

[208] Zalewski P.D., Forbes I.J., Betts W.H. (1993). Correlation of apoptosis with change in intracellular labile Zn(II) using zinquin [(2-methyl-8-p-toluenesulphonamido-6-quinolyloxy)acetic acid], a new specific fluorescent probe for Zn(II). Biochem. J..

[209] Williams R.J.P.A. (1989). Introduction to the Biochemistry of Zinc.

[210] Palmiter R.D., Cole T.B., Findley S.D. (1996). ZnT-2, a mammalian protein that confers resistance to zinc by facilitating vesicular sequestration. EMBO J..

[211] Choi D., Yokoyama M., Koh J. (1988). Zinc neurotoxicity in cortical cell culture. Neuroscience.

[212] Provinciali M., Di Stefano G., Fabris N. (1995). Dose-dependent opposite effect of zinc on apoptosis in mouse thymocytes. Int. J. Immunopharmacol..

[213] Telford W.G., Fraker P.J. (1995). Preferential induction of apoptosis in mouse CD4+CD8+ alpha beta TCRloCD3 epsilon lo thymocytes by zinc. J. Cell Physiol..

[214] Yokoyama M., Koh J., Choi D. (1986). Brief exposure to zinc is toxic to cortical neurons. Neurosci. Lett..

[215] Palmiter R. (1995). Constitutive Expression of Metallothionein-III (MT-III), but Not MT-I, Inhibits Growth When Cells Become Zinc Deficient. Toxicol. Appl. Pharm..

[216] Palmiter R.D. (2004). Protection against zinc toxicity by metallothionein and zinc transporter. Proc. Natl. Acad. Sci. USA.

[217] Palmiter R.D., Cole T.B., Quaife C.J., Findley S.D. (1996). ZnT-3, a putative transporter of zinc into synaptic vesicles. Proc. Natl. Acad. Sci. USA.

[218] Palmiter R., Findley S. (1995). Cloning and functional characterization of a mammalian zinc transporter that confers resistance to zinc. Embo J..

[219] Cuajungco M.P., Lees G.J. (1997). Zinc Metabolism in the Brain: Relevance to Human Neurodegenerative Disorders. Neurobiol. Dis..

[220] Maret W. (2017). Zinc in Cellular Regulation: The Nature and Significance of “Zinc Signals”. Int. J. Mol. Sci.

[221] McMahon R.J., Cousins R.J. (1998). Mammalian Zinc Transporters. J. Nutr..

[222] Howell G.A., Welch M.G., Frederickson C.J. (1984). Stimulation-induced uptake and release of zinc in hippocampal slices. Nat. Cell Biol..

[223] Kambe T., Tsuji T., Hashimoto A., Itsumura N. (2015). The Physiological, Biochemical, and Molecular Roles of Zinc Transporters in Zinc Homeostasis and Metabolism. Physiol. Rev..

[224] Aniksztejn L., Charton G., Ben-Ari Y. (1987). Selective release of endogenous zinc from the hippocampal mossy fibers in situ. Brain Res..

[225] Assaf S.Y., Chung S.-H. (1984). Release of endogenous Zn^2+^ from brain tissue during activity. Nat. Cell Biol..

[226] Charton G., Rovira C., Ben-Ari Y., Leviel V. (1985). Spontaneous and evoked release of endogenous Zn^2+^ in the hippocampal mossy fiber zone of the rat in situ. Exp. Brain Res..

[227] Bin B.H., Fukada T., Hosaka T., Yamasaki S., Ohashi W., Hojyo S., Miyai T., Nishida K., Yokoyama S., Hirano T. (2011). Biochemical characterization of human ZIP13 protein: A homo-dimerized zinc transporter involved in the spondylocheiro dysplastic Ehlers-Danlos syndrome. J. Biol. Chem..

[228] Kukic I., Lee J.K., Coblentz J., Kelleher S.L., Kiselyov K. (2013). Zinc-dependent lysosomal enlargement in TRPML1-deficient cells involves MTF-1 transcription factor and ZnT4 (Slc30a4) transporter. Biochem. J..

[229] Raffaniello R.D., Wapnir R.A. (1991). Zinc-induced metallothionein synthesis by Caco-2 cells. Biochem. Med. Metab. Biol..

[230] Thakran P., Leuschen M.P., Ebadi M. (1989). Metallothionein induction in rat hippocampal neurons in primary culture. Vivo.

[231] Valentine R.A., Jackson K.A., Christie G.R., Mathers J.C., Taylor P.M., Ford D. (2007). ZnT5 variant B is a bidirectional zinc transporter and mediates zinc uptake in human intestinal Caco-2 cells. J. Biol. Chem..

[232] Cuajungco M.P., Lees G.J. (1998). Nitric oxide generators produce accumulation of chelatable zinc in hippocampal neuronal perikarya. Brain Res..

[233] Jacob C., Maret W., Vallee B.L. (1998). Control of zinc transfer between thionein, metallothionein, and zinc proteins. Proc. Natl. Acad. Sci. USA.

[234] Jiang L.-J., Maret W., Vallee B.L. (1998). The glutathione redox couple modulates zinc transfer from metallothionein to zinc-depleted sorbitol dehydrogenase. Proc. Natl. Acad. Sci. USA.

[235] Wensink J., Molenaar A.J., Woroniecka U.D., Van den Hamer C.J. (1988). Zinc uptake into synaptosomes. J. Neurochem..

[236] Colvin R.A., Holmes W.R., Fontaine C.P., Maret W. (2010). Cytosolic zinc buffering and muffling: Their role in intracellular zinc homeostasis. Metallomics.

[237] Cousins R.J., Liuzzi J.P., Lichten L.A. (2006). Mammalian Zinc Transport, *Traffic*king, and Signals. J. Biol. Chem..

[238] Eide D.J. (2006). Zinc transporters and the cellular *Traffic*king of zinc. Biochim. Et Biophys. Acta (Bba) Bioenerg..

[239] Cuajungco M.P., Basilio L.C., Silva J., Hart T., Tringali J., Chen C.C., Biel M., Grimm C. (2014). Cellular Zinc Levels Are Modulated by TRPML1-TMEM163 Interaction. Traffic.

[240] Waberer L., Henrich E., Peetz O., Morgner N., Dotsch V., Bernhard F., Volknandt W. (2017). The synaptic vesicle protein SV31 assembles into a dimer and transports Zn(2). J. Neurochem..

[241] Sanchez V.B., Ali S., Escobar A., Cuajungco M.P. (2019). Transmembrane 163 (TMEM163) protein effluxes zinc. Arch. Biochem. Biophys..

[242] Ohana E., Hoch E., Keasar C., Kambe T., Yifrach O., Hershfinkel M., Sekler I. (2009). Identification of the Zn^2+^ binding site and mode of operation of a mammalian Zn^2+^ transporter. J. Biol. Chem..

[243] Maret W. (2008). Metallothionein redox biology in the cytoprotective and cytotoxic functions of zinc. Exp. Gerontol..

[244] Krezoski S.K., Villalobos J., Shaw C.F., Petering D.H. (1988). Kinetic lability of zinc bound to metallothionein in Ehrlich cells. Biochem. J..

[245] Beaulieu C., Dyck R., Cynader M. (1992). Enrichment of glutamate in zinc-containing terminals of the cat visual cortex. NeuroReport.

[246] Frederickson C.J., Klitenick M.A., Manton W.I., Kirkpatrick J.B. (1983). Cytoarchitectonic distribution of zinc in the hippocampus of man and the rat. Brain Res..

[247] Haug F.M. (1967). Electron microscopical localization of the zinc in hippocampal mossy fibre synapses by a modified sulfide silver procedure. Histochem. Cell Biol..

[248] Pe´rez-Clausell J., Danscher G. (1985). Intravesicular localization of zinc in rat telencephalic boutons. A histochemical study. Brain Res..

[249] Bresink I., Ebert B., Parsons C.G., Mutschler E. (1996). Zinc changes AMPA receptor properties: Results of binding studies and patch clamp recordings. Neuropharmacology.

[250] Christine C., Choi D. (1990). Effect of zinc on NMDA receptor-mediated channel currents in cortical neurons. J. Neurosci..

[251] Dreixler J.C., Leonard J.P. (1994). Subunit-specific enhancement of glutamate receptor responses by zinc. Mol. Brain Res..

[252] Xie X., Gerber U., Gahwiler B.H., Smart T.G. (1993). Interaction of zinc with ionotropic and metabotropic glutamate receptors in rat hippocampal slices. Neurosci. Lett..

[253] Yin H.Z., Weiss J.H. (1995). Zn^2+^ permeates Ca^2+^permeable AMPA/kainate channels and triggers selective neural injury. NeuroReport.

[254] Legendre P., Westbrook G.L. (1991). Noncompetitive inhibition of gamma-aminobutyric acidA channels by Zn. Mol. Pharm..

[255] Smart T.G., Moss S.J., Xie X., Huganir R.L. (1991). GABAA receptors are differentially sensitive to zinc: Dependence on subunit composition. Br. J. Pharmacol..

[256] Mayer M.L., Vyklicky L., Westbrook G.L. (1989). Modulation of excitatory amino acid receptors by group IIB metal cations in cultured mouse hippocampal neurones. J. Physiol..

[257] Mayer M.L., Vyklicky L. (1989). The action of zinc on synaptic transmission and neuronal excitability in cultures of mouse hippocampus. J. Physiol..

[258] Cloues R. (1995). Properties of ATP-gated channels recorded from rat sympathetic neurons: Voltage dependence and regulation by Zn^2+^ ions. J. Neurophysiol..

[259] A Connor M., Chavkin C. (1992). Ionic zinc may function as an endogenous ligand for the haloperidol-sensitive sigma 2 receptor in rat brain. Mol. Pharm..

[260] Kumamoto E., Murata Y. (1996). Glycine current in rat septal cholinergic neuron in culture: Monophasic positive modulation by Zn^2+^. J. Neurophysiol..

[261] Fabris N., Mocchegiani E. (1995). Zinc, human diseases and aging. Aging Clin. Exp. Res..

[262] Hexum T.D. (1974). Studies on the reaction catalyzed by transport (Na, K) adenosine triphosphatase—I. Biochem. Pharm..

[263] Hogstrand C., Verbost P.M., Wendelaar Bonga S.E. (1999). Inhibition of human erythrocyte Ca^2+^-ATPase by Zn^2+^. Toxicology.

[264] Link T.A., von Jagow G. (1995). Zinc ions inhibit the QP center of bovine heart mitochondrial bc1 complex by blocking a protonatable group. J. Biol. Chem..

[265] Wilson M., Hogstrand C., Maret W. (2012). Picomolar concentrations of free zinc(II) ions regulate receptor protein-tyrosine phosphatase beta activity. J. Biol. Chem..

[266] Hubbard S.R., Bishop W.R., Kirschmeier P., George S.J., Cramer S.P., Hendrickson W.A. (1991). Identification and characterization of zinc binding sites in protein kinase C. Science.

[267] Perry D.K., Smyth M.J., Stennicke H.R., Salvesen G.S., Duriez P., Poirier G.G., Hannun Y.A. (1997). Zinc is a potent inhibitor of the apoptotic protease, caspase-A novel target for zinc in the inhibition of apoptosis. J. Biol. Chem..

[268] Velázquez-Delgado E.M., Hardy J.A. (2012). Zinc-mediated Allosteric Inhibition of Caspase. J. Biol. Chem..

[269] Posewitz M.C., Wilcox D.E. (1995). Properties of the Sp1 Zinc Finger 3 Peptide: Coordination Chemistry, Redox Reactions, and Metal Binding Competition with Metallothionein. Chem. Res. Toxicol..

[270] Piątek K., Hartwig A., Bal W. (2009). Physiological levels of glutathione enhance Zn(II) binding by a Cys4 zinc finger. Biochem. Biophys. Res. Commun..

[271] Maret W. (1994). Oxidative metal release from metallothionein via zinc-thiol/disulfide interchange. Proc. Natl. Acad. Sci. USA.

[272] Baudier J., Haglid K., Haiech J., Gerard D. (1983). Zinc ion binding to human brain calcium binding proteins, calmodulin and S100b protein. Biochem. Biophys. Res. Commun..

[273] Eagle G.R., Zombola R.R., Himes R.H. (1983). Tubulin-zinc interactions: Binding and polymerization studies. Biochemistry.

[274] Cuajungco M.P., Lees G.J. (1997). Zinc and Alzheimer’s disease: Is there a direct link?. Brain Res..

[275] Donaldson J., St-Pierre T., Minnich J., Barbeau A. (1971). Seizures in Rats Associated with Divalent Cation Inhibition of Na+–K+-ATP’ase. Can. J. Biochem..

[276] Zhang G.H., Yamaguchi M., Kimura S., Higham S., Kraus-Friedmann N. (1990). Effects of heavy metal on rat liver microsomal Ca2(+)-ATPase and Ca^2+^ sequestering. Relation to SH groups. J. Biol. Chem..

[277] Lees G., Leong W. (1994). Brain lesions induced by specific and non-specific inhibitors of sodium-potassium ATPase. Brain Res..

[278] Lees G., Leong W. (1995). The sodium-potassium ATPase inhibitor ouabain is neurotoxic in the rat substantia nigra and striatum. Neurosci. Lett..

[279] Kleiner D., Von Jagow G. (1972). On the inhibition of mitochondrial electron transport by Zn^2+^ ions. FEBS Lett..

[280] Skulachev V.P., Chistyakov V.V., Jasaitis A.A., Smirnova E.G. (1967). Inhibition of the respiratory chain by zinc ions. Biochem. Biophys. Res. Commun..

[281] Gabrielsson B., Robson T., Norris D., Chung S.H. (1986). Effects of divalent metal ions on the uptake of glutamate and GABA from synaptosomal fractions. Brain Res..

[282] Velasco I., Tapia R., Massieu L. (1996). Inhibition of glutamate uptake induces progressive accumulation of extracellular glutamate and neuronal damage in rat cortical cultures. J. Neurosci. Res..

[283] Shen Z., Haragopal H., Li Y.V. (2020). Zinc modulates synaptic transmission by differentially regulating synaptic glutamate homeostasis in hippocampus. Eur. J. Neurosci..

[284] Maret W. (2013). Inhibitory zinc sites in enzymes. Biometals.

[285] Heizmann C.W., Cox A.J. (1998). New perspectives on S100 proteins: A multi-functional Ca 2+ -, Zn 2+ - and Cu 2+ -binding protein family. Biometals.

[286] Moroz O.V., Burkitt W., Wittkowski H., He W., Ianoul A., Novitskaya V., Xie J., Polyakova O., Lednev I.K., Shekhtman A. (2009). Both Ca^2+^ and Zn^2+^ are essential for S100A12 protein oligomerization and function. BMC Biochem..

[287] Moroz O.V., Wilson K.S., Bronstein I.B. (2010). The role of zinc in the S100 proteins: Insights from the X-ray structures. Amino Acids.

[288] Nickolson V., Veldstra H. (1972). The influence of various cations on the binding of colchicine by rat brain homogenates. Stabilization of intact neurotubules by zinc and cadmium ions. FEBS Lett..

[289] Kress Y., Gaskin F., Brosnan C.F., Levine S. (1981). Effects of zinc on the cytoskeletal proteins in the central nervous system of the rat. Brain Res..

[290] Hedberg K.K., Birrell G.B., Mobley P.L., Griffith O.H. (1994). Transition metal chelator TPEN counteracts phorbol ester-induced actin cytoskeletal disruption in C6 rat glioma cells without inhibiting activation or translocation of protein kinase C. J. Cell. Physiol..

[291] Kerr J.F., Wyllie A.H., Currie A.R. (1972). Apoptosis: A basic biological phenomenon with wide-ranging implications in tissue kinetics. Br. J. Cancer..

[292] Xu J., Xu Y., Nguyen Q., Novikoff P.M., Czaja M.J. (1996). Induction of hepatoma cell apoptosis by c-myc requires zinc and occurs in the absence of DNA fragmentation. Am. J. Physiol. Content.

[293] Xie Y., Hou W., Song X., Yu Y., Huang J., Sun X., Kang R., Tang D. (2016). Ferroptosis: Process and function. Cell Death Differ..

[294] Yang W.S., Stockwell B.R. (2016). Ferroptosis: Death by Lipid Peroxidation. Trends Cell Biol..

[295] Zalewski P., Forbes I., Seamark R., Borlinghaus R., Betts W., Lincoln S., Ward A. (1994). Flux of intracellular labile zinc during apoptosis (gene-directed cell death) revealed by a specific chemical probe, Zinquin. Chem. Biol..

[296] Fliss H., Ménard M. (1991). Hypochlorous acid-induced mobilization of zinc from metalloproteins. Arch. Biochem. Biophys..

[297] Fliss H., Ménard M. (1992). Oxidant-induced mobilization of zinc from metallothionein. Arch. Biochem. Biophys..

[298] Fliss H., Ménard M., Desai M. (1991). Hypochlorous acid mobilizes cellular zinc. Can. J. Physiol. Pharm..

[299] Baba A., Kihara T., Sawada T., Iwata H. (1989). Excitatory amino acids enhance dissociation of zinc from soluble protein in cytosol of rat hippocampus. Brain Res..

[300] Colletti G.A., Miedel M.T., Quinn J.S., Andharia N., Weisz O.A., Kiselyov K. (2012). Loss of Lysosomal Ion Channel Transient Receptor Potential Channel Mucolipin-1 (TRPML1) Leads to Cathepsin B-dependent Apoptosis. J. Biol. Chem..

[301] Venkatachalam K., Long A.A., Elsaesser R., Nikolaeva D., Broadie K., Montell C. (2008). Motor Deficit in a Drosophila Model of Mucolipidosis Type IV due to Defective Clearance of Apoptotic Cells. Cell.

[302] Yu H., Zhou Y., Lind S.E., Ding W.-Q. (2008). Clioquinol targets zinc to lysosomes in human cancer cells. Biochem. J..

[303] Chacon J., Rosas L., Cuajungco M.P. (2019). ZnT3 expression levels are down-regulated in the brain of Mcoln1 knockout mice. Mol. Brain.

[304] Koh J.-Y., Suh S.W., Gwag B.J., He Y.Y., Hsu C.Y., Choi D.W., Novelli M.R., Williamson J.A., Tomlinson I.P.M., Elia G. (1996). The Role of Zinc in Selective Neuronal Death After Transient Global Cerebral Ischemia. Science.

[305] Frederickson C., Hernández M., McGinty J. (1989). Translocation of zinc may contribute to seizure-induced death of neurons. Brain Res..

[306] Charriaut-Marlangue C., Aggoun-Zouaoui D., Represa A., Ben-Ari Y. (1996). Apoptotic features of selective neuronal death in ischemia, epilepsy and gpI20 toxicity. Trends Neurosci..

[307] Arslan P., Di Virgilio F., Beltrame M., Tsien R.Y., Pozzan T. (1985). Cytosolic Ca^2+^ homeostasis in Ehrlich and Yoshida carcinomas. A new, membrane-permeant chelator of heavy metals reveals that these ascites tumor cell lines have normal cytosolic free Ca^2+^. J. Biol. Chem..

[308] May P.M., Bulman R.A. (1983). 5 The Present Status of Chelating Agents in Medicine. Prog. Med. Chem..

[309] Mellor D. (1964). Historical Background and Fundamental Concepts.

[310] Gibson W., Hardy W., Groom M. (1985). The effect and mode of action of zinc pyrithione on cell growth. II. in vivo studies. Food Chem. Toxicol..

[311] Gibson W., Chamberlain M., Parsons J., Brunskill J., Leftwich D., Lock S., Safford R. (1985). The effect and mode of action of zinc pyrithione on cell growth. I. in vitro studies. Food Chem. Toxicol..

[312] Park M.-H., Lee S.-J., Byun H.-R., Kim Y., Oh Y.J., Koh J.-Y., Hwang J.J. (2011). Clioquinol induces autophagy in cultured astrocytes and neurons by acting as a zinc ionophore. Neurobiol. Dis..

[313] Hider R., Hall A. (1991). 2 Clinically Useful Chelators of Tripositive Elements. Prog. Med. Chem..

[314] Cuajungco M.P., Lees G.J. (1998). Diverse effects of metal chelating agents on the neuronal cytotoxicity of zinc in the hippocampus. Brain Res..

[315] Anderegg G., Hubmann E., Podder N.G., Wenk F. (1977). Pyridine derivatives as complexing agents XI: Thermodynamics of metal complex formation with bis-, tris-, and tetrakis[(2-pyridyl)methyl]-amines. Helv. Chim. Acta.

[316] Treves S., Trentini P.L., Ascanelli M., Bucci G., Di Virgilio F. (1994). Apoptosis Is Dependent on Intracellular Zinc and Independent of Intracellular Calcium in Lymphocytes. Exp. Cell Res..

[317] A Lazebnik Y., Cole S., A Cooke C., Nelson W.G., Earnshaw W.C. (1993). Nuclear events of apoptosis in vitro in cell-free mitotic extracts: A model system for analysis of the active phase of apoptosis. J. Cell Biol..

[318] Jiang S., Chow S.C., McCabe M.J., Orrenius S. (1995). Lack of Ca^2+^ involvement in thymocyte apoptosis induced by chelation of intracellular Zn^2+^. Lab. Investig..

[319] Cuajungco M.P., Lees G.J. (1996). Prevention of zinc neurotoxicity in vivo by N,N,N’,N’-tetrakis (2-pyridylmethyl) ethylene-diamine (TPEN). Neuroreport.

[320] Choi D.W., Koh J.Y. (1998). ZINC AND BRAIN INJURY. Annu. Rev. Neurosci..

[321] Choi D.W., Weiss J.H., Koh J.-Y., Christine C.W., Kurth M.C. (1989). Glutamate Neurotoxicity, Calcium, and Zinc. Ann. N. Y. Acad. Sci..

